# Plant-Based Diets, Ultra-Processed Foods, and Risks of Mortality and Major Chronic Diseases: A Prospective Cohort Study

**DOI:** 10.1016/j.lanepe.2026.101736

**Published:** 2026-06-05

**Authors:** Alysha S. Thompson, Amy Jennings, Nicola P. Bondonno, Aedín Cassidy, Tilman Kühn

**Affiliations:** a Co-Centre for Sustainable Food Systems and The Institute for Global Food Security, Queen's University Belfast, Northern Ireland, UK; b Danish Cancer Institute, Copenhagen, Denmark; c Nutrition & Health Innovation Research Institute, School of Medical and Health Sciences, Edith Cowan University, Joondalup, WA, Australia; d Department of Nutritional Sciences, University of Vienna, Vienna, Austria; e Center for Public Health, Medical University of Vienna, Vienna, Austria; f Department of Nutritional, Food and Consumer Sciences, Fulda University of Applied Sciences, Fulda, Germany

**Keywords:** Plant-based diets, Ultra-processed foods, Nutritional quality, Mortality, Chronic disease

## Abstract

**Background:**

Higher-quality plant-based diets (PBDs) are associated with lower risks of mortality and chronic disease, but whether ultra-processed food (UPF) content affects these associations remains unclear. We examined whether UPF content influences the relationship between plant-based dietary patterns and risks of mortality and major chronic diseases, accounting for nutrient quality.

**Methods:**

This prospective cohort study included 124,836 UK Biobank participants aged 40–70 years (recruited 2006–2010). Dietary intake was assessed using the Oxford WebQ 24-h recall. Four modified Plant-Based Diet Indices (PDIs) were derived to distinguish healthy (hPDI) and unhealthy (uPDI) patterns with high- and low-UPF content, using the Nova classification and a Modified Nutrient Quality Index (mNQI). Participants were followed for 8.3–10.5 years for all-cause mortality and incident T2DM, CVD, and cancer. Multivariable Cox models estimated hazard ratios (HRs) and 95% confidence intervals (CIs).

**Findings:**

Among 124,836 participants (mean [SD] age 56.2 [7.8] years; 55.8% women), there were 5780 deaths, 3420 T2DM cases, 6078 CVD cases, and 9437 cancer cases. Higher adherence to healthy plant-based diets—whether high- or low-UPF—was associated with 8–28% lower risk of all-cause mortality [HR_Q4vsQ1_ (95% CI): high-UPF hPDI, 0.92 (0.85–1.00); low-UPF hPDI, 0.91 (0.84–0.98)] and type 2 diabetes [high-UPF hPDI, 0.89 (0.79–0.99); low-UPF hPDI, 0.72 (0.65–0.79)]. Higher adherence to the high-UPF hPDI was also associated with 11% lower cardiovascular disease risk [0.89 (0.82–0.96)], while no clear association was observed for the low-UPF hPDI. Nutrient quality was similar across high- and low-UPF hPDI patterns.

**Interpretation:**

Adherence to healthful PBDs is associated with more favourable health outcomes irrespective of UPF content, suggesting that overall PBD quality may be more important than processing level for chronic disease prevention.

**Funding:**

This research was supported by Research Ireland, Northern Ireland's Department of Agriculture, Environment and Rural Affairs (DAERA), UK Research and Innovation (UKRI) via the International Science Partnerships Fund (ISPF) under Grant number 22/CC/11147 at the Co–Centre for Sustainable Food Systems.

**Research in context**

**Evidence before this study**

We searched PubMed from database inception to October 30, 2025, for studies examining the relationship between plant-based dietary patterns, ultra-processed food (UPF) consumption, and risks of mortality and major chronic diseases. We used combinations of search terms including “plant-based diet”, “plant-based dietary pattern”, “plant-based diet index”, “healthy plant-based diet”, “ultra-processed food”, “Nova”, “diet quality”, “mortality”, “cardiovascular disease”, “type 2 diabetes”, and “cancer”, with no language restrictions. Previous evidence consistently shows that higher adherence to healthy plant-based diets is associated with lower risk of chronic disease and mortality, whereas unhealthy plant-based diets are linked to poorer outcomes. Only a small number of cohort studies have examined the role of UPF content within plant-based diets. The NutriNet-Santé cohort investigated cardiovascular disease risk in relation to UPF content in plant-based diets in a French population, while the EPIC-NL study assessed associations with all-cause mortality and environmental outcomes. However, no studies to date have examined whether UPF content affects the associations between plant-based dietary patterns and multiple major chronic diseases simultaneously, while accounting for nutrient quality.

**Added value of this study**

Using data from 124,836 UK Biobank participants followed for up to 10.5 years, we derived four modified Plant-Based Diet Indices to distinguish healthy (hPDI) and unhealthy (uPDI) patterns with high- and low-UPF content, integrating both Nova classification and a modified nutrient-quality index. This is the first large-scale study to evaluate how UPF content within plant-based diets influences risks of all-cause mortality, type 2 diabetes, cardiovascular disease, and cancer in the same population. We demonstrate that higher adherence to healthful plant-based diets is associated with lower risks of mortality, type 2 diabetes, and cardiovascular disease, regardless of UPF content. These findings challenge the prevailing narrative that UPFs are universally harmful.

**Implications of all the available evidence**

Taken together with prior studies, our findings suggest that public health recommendations should move beyond a focus on processing level and instead prioritise the nutritional quality of plant-based dietary patterns. While some UPFs may be detrimental, others with favourable nutrient profiles may form part of a healthful diet when embedded within an overall high-quality dietary pattern. Future research should explore more diverse populations, longer-term trajectories, and mechanistic pathways to better inform dietary guidance and policy on different types of UPFs.

## Introduction

Food production drives around one-third of global greenhouse gas emissions and strains land, water, and biodiversity.^1^^,^^2^ Diets high in animal-sourced foods, particularly red and processed meats, are environmentally burdensome^3^^,^^4^ and linked to greater risks of mortality, cardiometabolic disease and certain cancers.^5^, ^6^, ^7^ Consequently, global dietary guidelines, including the EAT-Lancet Commission report, encourage a shift towards more plant-based diets (PBDs).^8^

Many modern PBDs include industrially formulated meat and dairy alternatives classified as ultra-processed foods (UPFs).^9^ UPFs are often characterised as energy-dense, nutrient-poor, and engineered for palatability,^9^ and although often marketed as sustainable or healthful, some plant-based analogues may undermine the benefits of PBDs. In the UK and US, UPFs already account for more than half of total dietary energy.^10^ Large scale cohort studies and recent comprehensive syntheses consistently demonstrate that higher UPF consumption is associated with greater risks of death and major chronic disease,^11^^,^^12^ and randomised trials suggest that reducing UPF intake promotes weight loss via lower energy consumption.^13^^,^^14^ Thus, policy measures to reduce UPF consumption have been proposed.^15^

Despite concerns about adverse health effects of UPFs, the UPF concept itself has faced criticism. Not all UPFs are nutritionally poor and reducing them indiscriminately could discourage nutrient-dense products such as wholegrain breads or fortified foods. Broad recommendations to limit UPFs may oversimplify dietary guidance and divert attention from overall diet quality. Recent evidence suggests that vegetarians and vegans may consume more UPFs than omnivores, driven by growing consumption of plant-based substitutes.^16^^,^^17^ Yet, little is known about how differences in UPF content within PBDs influence long-term health outcomes, particularly when nutritional quality is also considered.

Given the lack of studies on UPFs in PBDs, we used UK Biobank data to examine how UPF content modifies associations between plant-based dietary patterns and mortality and chronic disease risk, accounting for nutrient quality. Four modified Plant-Based Diet Indices (PDIs) were developed, differentiating high- and low-UPF content using Nova to clarify whether the benefits of PBDs are driven by nutrient quality or degree of processing.

## Methods

### Study population

This UK Biobank is a large population-based cohort of >500,000 adults (aged 40–69 years) recruited in 2006–2010 across 22 centres in England, Scotland, and Wales. Participants completed touchscreen questionnaires, physical measures, and provided biological samples. All UK Biobank participants provided written informed consent for participation and follow-up through linkage to health records. The UK Biobank study received ethical approval from the NHS North West Multicentre Research Ethics Committee (Ref. 11/NW/0382).

**Fig. 1:**
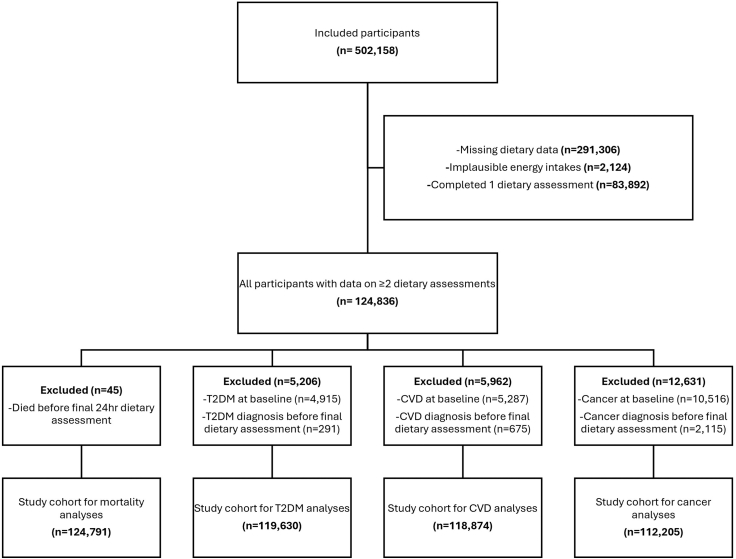
**Flowchart of exclusion**.

Of 502,158 participants, we excluded those with withdrawn consent, missing dietary data (n = 291,306), implausible energy intakes (>4200 or <800 kcal/day for men; >3500 or <500 kcal/day for women^18^; n = 2124), or <2 valid 24-h dietary assessments (n = 83,892), leaving 124,836 participants. For each outcome, participants with a history of the relevant condition (type 2 diabetes (T2DM), cardiovascular disease (CVD), or cancer) before their last recorded dietary assessment, identified via hospital records or self-reported data, were also excluded (Fig. 1).

### Dietary assessment and exposure definition

Diet was assessed using the Oxford WebQ,^19^^,^^20^ a validated web-based 24-h dietary assessment tool,^21^^,^^22^ administered once at baseline (2009–2010), and up to four times online (2011–2012). To improve estimation of habitual intake and reduce within-person variability, analyses were restricted to participants who completed at least two valid 24-h dietary assessments. Dietary exposures were derived using the mean intake across all available recalls. Among the 124,836 included participants, 38.7% completed two dietary recalls, 33.6% completed three, 23.4% completed four, and 4.3% completed five recalls.

We developed four modified versions of the healthful (hPDI) and unhealthful (uPDI) PDIs by incorporating food processing level using the Nova classification. Nova categorises foods according to the extent and purpose of industrial processing: ultra-processed foods (UPFs; Nova Group 4) are industrial formulations that are typically energy-dense, nutrient-poor, and engineered for palatability, whereas non-UPFs (Nova Groups 1–3) include unprocessed, minimally processed, and processed culinary ingredients.^9^ Each Oxford WebQ food item was classified according to Nova based on previously published UK Biobank studies,^23^^,^^24^ allowing us to distinguish high- and low-UPF foods within the PDI food groups originally defined by Satija et al.^25^ The original PDIs comprise 17 food groups, categorised as healthy plant foods, less healthy plant foods, or animal foods, with positive or reverse scoring applied accordingly. Each food group was disaggregated to the individual food-item level using Oxford WebQ data, and each item was classified as either ultra-processed (Nova Group 4) or non-ultra-processed (Nova Groups 1–3). These classifications were used to construct four distinct dietary indices, all derived from the same underlying 17–food-group PDI framework and scored according to the original PDI rules (i.e., healthy plant foods positively for hPDI, less healthy plant foods positively for uPDI, and other food groups in reverse). The four indices differed in the processing level of foods contributing to each food group: High-UPF hPDI (UPFs only), Low-UPF hPDI (non-UPFs only), High-UPF uPDI (UPFs only), and Low-UPF uPDI (non-UPFs only) (Supplementary Table S1). For each index, only foods within the relevant processing category contributed to the score, while foods outside that category were ignored. As a result, some food groups did not contribute to certain index variants when no eligible foods were present within the relevant processing category (e.g., fruit intake was excluded from the high-UPF hPDI, while sugar-sweetened beverages (SSBs) were excluded from the low-UPF hPDI). Vegetable oils, included in previous PDI constructions, were not incorporated due to data unavailability in the UK Biobank.^26^ Participants were scored for each food group based on average daily portion intake. For positively scored food groups, non-consumers received a score of 1, while remaining participants were ranked into sex-specific quartiles and assigned scores from 2 (lowest intake) to 5 (highest intake). For reverse scored food groups, this scoring was inverted. Final index scores were calculated by summing scores across all contributing food groups, with higher scores indicating greater adherence to the respective dietary pattern. Each PDI was calculated as a continuous score, with higher values indicating greater adherence to the respective dietary pattern. Each PDI was calculated as a continuous score, resulting in four separate scores for each participant: high-UPF hPDI, low-UPF hPDI, high-UPF uPDI, and low-UPF uPDI. An overview of the classification of Oxford WebQ food items into PDI food groups by Nova processing level is provided in Supplementary Table S1, and a visual overview of the 17 food groups, their UPF classification, and scoring in the four modified PDIs is presented in Supplementary Figure S1.

A Modified Nutrient Quality Index (mNQI) was derived using average intakes of five nutrients obtained from the Oxford WebQ: two positive components (dietary fibre and protein) and three negative components (saturated fat, total sugars, and salt). Intakes of each nutrient were categorised into quartiles. Fibre and protein were positively scored (higher quartiles receiving higher scores), while saturated fat, total sugars, and salt were reverse scored (higher quartiles receiving lower scores). Component scores were summed to generate a total mNQI score, with higher values indicating better overall nutrient quality (i.e., higher fibre and protein and lower saturated fat, sugar, and salt).

### Case ascertainment and covariate assessment

Outcomes were all-cause mortality and incident T2DM, CVD, and cancer. Mortality data were obtained through linkage with national death registries: the NHS information Centre for England and Wales, and the NHS Central Register for Scotland. Hospital admissions and diagnoses were ascertained via linkage to Health Episode Statistics (England), the Patient Episode Database for Wales, and the Scottish Morbidity Records (Scotland), as well as national cancer registries across all three nations. Incident outcomes were defined as the first occurrence of a hospital admission or death recorded under relevant primary or secondary diagnosis codes, using he International Classification of Diseases, 10th Revision (ICD-10). CVD was identified using the following codes: any CVD (I20–I23, I24.1, I25.2 I60–I61, I63, I64). T2DM was defined using E11. Cancer outcomes included any cancer (C00–C97, excluding non-melanoma skin cancer [C44]).

Follow-up for hospital admissions related to T2DM, and CVD was available until 31 October 2022 for England, 31 August 2022 for Scotland, and 31 May 2022 for Wales. Cancer registry data were available up to 31 December 2020 for England, 30 November 2021 for Scotland, and 31 December 2016 for Wales. Mortality data were available until 30 November 2022 for all nations. Outcome analyses were censored at the end of follow-up for each respective data source. Summary statistics for follow-up time across outcomes are provided in Supplementary Table S2.

At baseline (2006–2010), UK Biobank participants completed questionnaires capturing sociodemographic, dietary, and lifestyle information. Trained staff also obtained anthropometric measurements and collected biological samples from all participants. Additional details on the covariates used in this analysis are provided in Supplementary Methods S1 and Supplementary Table S3.

### Statistical analysis

Baseline characteristics were described overall and across extreme quartiles (Q1 vs Q4) of each index. For analytical analyses, PDI scores were modelled both as sex-specific quartiles to allow comparison across levels of adherence, and as continuous variables to assess linear trends (*P*-trend). Given potential overlap between indices, pairwise Pearson correlations were calculated (Supplementary Figure S2). Cox proportional hazards models estimated hazard ratios (HRs) and 95% CIs, with attained age (years) as the underlying timescale.^27^ Participants were followed from last valid dietary assessment until the first occurrence of the outcome, death, loss to follow-up, or end of follow-up, whichever came first. Models were adjusted sequentially: Model 1 included sociodemographic variables (sex, education, deprivation index, ethnicity) and was stratified by age group and region. Model 2 additionally adjusted for lifestyle and clinical factors (BMI, physical activity, smoking, alcohol, energy intake, multimorbidity, polypharmacy, relevant medications, and number of dietary assessments). Outcome-specific adjustments were applied for baseline comorbidities (CVD, T2DM, cancer) and, for cancer models, menopausal status and hormone therapy use. Covariates were selected based on prior evidence of confounding in diet–disease associations, including sociodemographic, lifestyle, and clinical factors.^28^, ^29^, ^30^, ^31^, ^32^, ^33^ For more details on covariate coding and categorisation, see Supplementary Table S3. Linear trends were tested by modelling PDIs continuously (P_trend_). To reduce data loss, missing or “unknown” responses (e.g., “prefer not to answer” or “do not know”) were retained via indicator categories. Models for high-UPF indices were additionally adjusted for food groups excluded from these scores due to lack of UPF content, including fruit, nuts, animal fat, fruit juice and eggs. Conversely, models for the low-UPF indices were additionally adjusted for food groups excluded because they contained only high-UPF components, including SSBs, sweets and desserts, and miscellaneous animal-based foods.

Multivariable adjusted models (Model 2) were applied to the 17 major PDI food groups, split by high- and low-UPF content, to assess their contribution to the observed associations. Sensitivity analyses assessed (1) influence of individual food groups (leave-one-out), (2) independence of associations from nutrient quality (mNQI adjustment), (3) the ratio of low-to high-UPF PDI scores, (4) potential modification of the association between the hPDI and uPDI by overall UPF intake (low < median vs. high ≥ median), using likelihood ratio tests for interaction, and (5) potential effect modification by sex, assessed through sex-stratified analyses for all outcomes. For all-cause mortality, additional sensitivity analyses included (6) exclusion of deaths occurring within one year of the last dietary assessment to minimise potential reverse causation, and (7) restriction of follow-up to events occurring before 1st January 2020 to account for the potential influence of the COVID-19 pandemic.

All statistical analyses were conducted using Stata version 18.5 (StataCorp LLC) and R statistics (v.4.5.1). Statistical significance was set at two-sided P < 0.05. Proportional hazards assumptions were verified using Schoenfeld residuals, with no violations detected for the variables of interest (p > 0.05). Cumulative hazard plots were generated for primary exposures. To ensure stable estimates when using attained age as the timescale, analyses were restricted to attained age <80 years.

### Role of the funding source

The funding organisations had no role in the design or conduct of the study; collection, management, analysis, or interpretation of the data; preparation, review, or approval of the manuscript; or the decision to submit the manuscript for publication.

## Results

### Characteristics of the study population

Of 502,158 UK Biobank participants, 124,836 with ≥2 dietary assessments were included. At baseline, the mean (SD) age was 56.2 (7.8) years, 55.8% were women, and 96.6% were White. Most participants were non-smokers (>90%), and 60% were overweight or obese. The mean (SD) scores for the four dietary indices were 37.0 (4.7) for the high-UPF hPDI, 45.1 (5.4) for the low-UPF hPDI, 44.0 (4.4) for the high-UPF uPDI, and 44.9 (5.3) for the low-UPF uPDI. During a mean follow-up ranging from 8.3–10.5 years across outcomes, 5780 deaths, 3420 T2DM cases, 6078 CVD cases, and 9437 cancer cases were recorded. Correlations between the four indices were generally weak; the strongest was a moderate inverse correlation between high-UPF hPDI and high-UPF uPDI (r = −0.50; Supplementary Figure S2), indicating that individuals with greater adherence to a healthful PBD characterised by UPFs tended, as expected, to have lower adherence to an unhealthful plant-based dietary pattern based on UPFs, although the two patterns were not mutually exclusive.

Included participants were slightly younger, leaner, and more often women, White, and non-smokers than those excluded (Supplementary Table S4). To illustrate the nutrient profiles underlying adherence to each PDI, Supplementary Tables S5–S8 present key nutrient intakes across quartiles of the high- and low-UPF variants of the hPDI and uPDI. Across all indices, participants in the highest hPDI quartile (Q4) exhibited more favourable nutrient profiles compared with those in the lowest quartile (Q1), including higher fibre and mNQI, and lower energy, total fat, saturated fat, and cholesterol. By contrast, higher adherence to the uPDI (Q4 vs Q1) was associated with poorer nutrient quality, characterised by lower fibre and mNQI and higher fat and cholesterol intakes, particularly for high-UPF variants. Differences between high- and low-UPF variants within each index were modest. In the ratio-based analyses, higher quartiles of the low-UPF:high-UPF hPDI were characterised by slightly higher fibre and calcium intakes and lower cholesterol, alongside higher total energy intake. For the low-UPF:high-UPF uPDI ratio, higher quartiles were associated with lower energy, total fat, saturated fat, and cholesterol intakes, with modest improvements in overall nutrient quality (Supplementary Tables S9–10).

As shown in Table 1, participants in the highest quartile (Q4) of the low-UPF hPDI were more likely to be female, older, more physically active, have lower BMI, and be never-smokers compared to those in the lowest quartile (Q1). For the high-UPF hPDI, Q4 participants were more likely to be male, older, more physically active, have a lower BMI, higher educational attainment, and fewer long-term health conditions or regular medications than Q1 participants.

**Table 1:** Baseline characteristics across quartiles of high-ultra-processed and low-ultra-processed healthful and unhealthful plant-based diet index (n = 124,791).

Characteristic	High-UPF hPDI		Low-UPF hPDI		High-UPF uPDI		Low-UPF uPDI		Whole sample
	Q1 (n = 37,692)	Q4 (n = 24,388)	Q1 (n = 32,136)	Q4 (n = 28,032)	Q1 (n = 36,207)	Q4 (n = 30,501)	Q1 (n = 35,354)	Q4 (n = 28,037)	(n = 124,791)
Sex-Female	22,213 (58.9)	13,123 (53.8)	18,298 (56.9)	16,893 (60.3)	21,421 (59.2)	17,057 (55.9)	19,316 (54.6)	15,984 (57.0)	69,664 (55.8)
Age at recruitment (years)	55.0 (8.0)	57.0 (7.5)	55.2 (8.0)	56.9 (7.5)	57.1 (7.5)	54.7 (8.1)	57.3 (7.5)	54.6 (8.1)	56.2 (7.8)
BMI (kg/m^2^)	27.6 (4.9)	25.9 (4.3)	27.2 (4.9)	26.1 (4.3)	26.3 (4.4)	27.4 (4.9)	26.8 (4.6)	26.8 (4.7)	26.7 (4.6)
Energy intake (kJ/day)	9158.3 (1955.1)	7942.9 (1849.9)	8921.4 (1940.5)	8273.9 (1947.5)	8444.9 (1919.6)	8779.4 (1984.0)	9169.2 (2001.8)	7919.1 (1843.6)	8545.2 (1959.1)
Physical activity (MET-h/wk)	29.8 (39.7)	32.8 (38.9)	28.4 (36.1)	34.6 (41.8)	32.1 (38.2)	30.1 (39.9)	34.5 (41.2)	27.9 (35.8)	31.1 (38.5)
Ethnicity									
Asian	386 (1.0)	332 (1.4)	322 (1.0)	408 (1.5)	385 (1.1)	360 (1.2)	286 (0.8)	473 (1.7)	1463 (1.2)
Black	282 (0.8)	207 (0.9)	320 (1.0)	196 (0.7)	180 (0.5)	338 (1.1)	176 (0.5)	356 (1.3)	965 (0.8)
Mixed	210 (0.6)	121 (0.5)	205 (0.6)	150 (0.5)	161 (0.4)	185 (0.6)	188 (0.5)	161 (0.6)	667 (0.5)
White	36,534 (96.9)	23,463 (96.2)	30,983 (96.4)	27,028 (96.4)	35,160 (97.1)	29,359 (96.3)	34,409 (97.3)	26,760 (95.5)	120,557 (96.6)
Other^a^	182 (0.5)	174 (0.7)	198 (0.6)	166 (0.6)	200 (0.6)	163 (0.5)	171 (0.5)	204 (0.7)	723 (0.6)
Education level									
Low	6343 (16.8)	2690 (11.0)	4277 (13.3)	3792 (13.5)	4439 (12.3)	5097 (16.7)	4387 (12.4)	4365 (15.6)	17,121 (13.7)
Medium	7345 (19.5)	3539 (14.5)	5521 (17.2)	4659 (16.6)	5486 (15.2)	5964 (19.6)	5644 (16.0)	5130 (18.3)	21,175 (17.0)
High	20,863 (55.4)	16,940 (69.5)	20,392 (63.5)	17,552 (62.6)	24,164 (66.7)	16,852 (55.3)	23,077 (65.3)	16,586 (59.2)	78,007 (62.5)
Townsend deprivation index									
Q1 (low deprivation)	7671 (20.4)	4809 (19.7)	6371 (19.8)	5808 (20.7)	7479 (20.7)	6024 (19.8)	7312 (20.7)	5571 (19.9)	25,726 (20.6)
Q2	7658 (20.3)	4624 (19.0)	6204 (19.3)	5759 (20.5)	7363 (20.3)	6108 (20.0)	7207 (20.4)	5475 (19.5)	25,042 (20.1)
Q3	7816 (20.7)	4666 (19.1)	6363 (19.8)	5758 (20.5)	7250 (20.0)	6278 (20.6)	7067 (20.0)	5653 (20.2)	25,145 (20.2)
Q4	7453 (19.8)	4990 (20.5)	6523 (20.3)	5536 (19.8)	7305 (20.2)	6005 (19.7)	7094 (20.1)	5640 (20.1)	24,891 (20.0)
Q5 (high deprivation)	7039 (18.7)	5276 (21.6)	6628 (20.6)	5141 (18.3)	6766 (18.7)	6036 (19.8)	6636 (18.8)	5661 (20.2)	23,841 (19.1)
Smoking status									
Never	21,933 (58.2)	13,600 (55.8)	18,156 (56.5)	16,198 (57.8)	21,065 (58.2)	17,145 (56.2)	19,093 (54.0)	17,142 (61.1)	71,314 (57.2)
Previous	12,752 (33.8)	9206 (37.8)	11,215 (34.9)	10,309 (36.8)	13,090 (36.2)	10,464 (34.3)	13,849 (39.2)	8805 (31.4)	44,632 (35.8)
Current	2925 (7.8)	1537 (6.3)	2713 (8.4)	1457 (5.2)	1972 (5.5)	2823 (9.3)	2337 (6.6)	2020 (7.2)	8578 (6.9)
Alcohol intake (g/day)	7.9 (9.4)	8.7 (10.0)	10.3 (10.9)	6.9 (8.5)	8.0 (9.3)	8.7 (10.3)	8.9 (9.8)	8.3 (9.9)	8.7 (9.9)
Multimorbidity, No. of long-term conditions									
0	14,353 (38.1)	10,165 (41.7)	13,014 (40.5)	10,790 (38.5)	14,547 (40.2)	11,755 (38.5)	13,560 (38.4)	11,632 (41.5)	49,490 (39.7)
1	12,560 (33.3)	8146 (33.4)	10,790 (33.6)	9477 (33.8)	12,167 (33.6)	10,126 (33.2)	11,927 (33.7)	9268 (33.1)	41,992 (33.7)
2	6649 (17.6)	3915 (16.1)	5258 (16.4)	4947 (17.7)	6039 (16.7)	5283 (17.3)	6198 (17.5)	4534 (16.2)	21,102 (16.9)
≥3	4130 (11.0)	2162 (8.9)	3074 (9.6)	2818 (10.1)	3454 (9.5)	3337 (10.9)	3669 (10.4)	2603 (9.3)	12,207 (9.8)
Polypharmacy, No. of medications									
0	10,885 (28.9)	8188 (33.6)	10,265 (31.9)	8388 (29.9)	11,308 (31.2)	9136 (30.0)	10,842 (30.7)	8796 (31.4)	38,689 (31.0)
1–3	17,717 (47.0)	11,239 (46.1)	15,076 (46.9)	13,008 (46.4)	16,819 (46.5)	14,240 (46.7)	16,189 (45.8)	13,161 (46.9)	58,184 (46.6)
4–6	6532 (17.3)	3697 (15.2)	4999 (15.6)	4883 (17.4)	5987 (16.5)	5145 (16.9)	6091 (17.2)	4457 (15.9)	20,557 (16.5)
7–9	1821 (4.8)	970 (4.0)	1296 (4.0)	1342 (4.8)	1564 (4.3)	1392 (4.6)	1656 (4.7)	1181 (4.2)	5413 (4.3)
≥10	729 (1.9)	291 (1.2)	493 (1.5)	409 (1.5)	525 (1.5)	582 (1.9)	568 (1.6)	436 (1.6)	1928 (1.5)
Medication use									
Blood pressure medication	7322 (19.4)	4181 (17.1)	5877 (18.3)	5096 (18.2)	6419 (17.7)	5931 (19.5)	6799 (19.2)	4913 (17.5)	23,228 (18.6)
Blood thinning medication	4864 (12.9)	3244 (13.3)	3962 (12.3)	3887 (13.9)	4725 (13.1)	3995 (13.1)	5101 (14.4)	3395 (12.1)	16,434 (13.2)
Cholesterol lowering medication	5674 (15.1)	3536 (14.5)	4521 (14.1)	4322 (15.4)	5174 (14.3)	4612 (15.1)	5481 (15.5)	3978 (14.2)	18,707 (15.0)

Data are expressed as mean (SD) or *n* (%), unless otherwise stated. Relative frequencies (%) include missing values which may not equate to 100%.

Abbreviations: UPF, ultra-processed food; Q, quartile; hPDI, healthful plant-based diet index; uPDI, unhealthful plant-based index; BMI, body mass index; MET, metabolic equivalent task; SD, standard deviation.

a Other includes any race or ethnic group not otherwise specified.

**Fig. 2:**
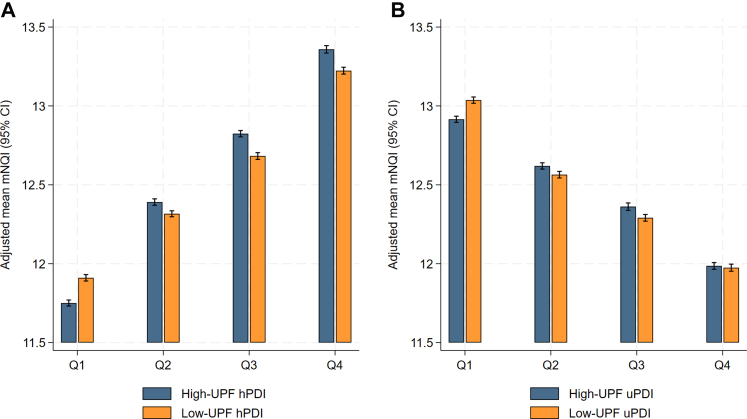
**Adjusted mean nutrient quality (mNQI, 95% CI) across quartiles (Q1–Q4) of: (A) hPDI and (B) uPDI, stratified by high- and low-UPF content.** Estimated marginal means were obtained from linear regression models with mNQI as the outcome, adjusting for sex, education, ethnicity, BMI, physical activity, smoking status, alcohol intake, energy intake, multimorbidity index, polypharmacy, blood pressure medications, blood thinning medications, cholesterol lowering medications, Townsend deprivation index, number of completed dietary assessments, age, and region. Abbreviations: Q, quartile; UPF, ultra-processed food; hPDI, healthful plant-based diet index; uPDI, unhealthful plant-based diet index; BMI, Body Mass Index; CI, confidence interval.

Dietary patterns differed across quartiles of the indices. Higher high-UPF hPDI scores were characterised by higher intakes of wholegrains and lower intakes of refined grains, potatoes, SSBs, sweets and desserts, and animal-based foods, whereas higher low-UPF hPDI scores reflected higher intakes of fruit, vegetables, nuts, legumes, tea and coffee, with less fruit juice and animal-based foods. In contrast, higher high-UPF uPDI scores were associated with higher intake of refined grains, potatoes, SSBs, and sweets and desserts and lower intakes of wholegrains and animal-based foods, while higher low-UPF uPDI scores reflected higher fruit juice intake but lower intakes of other plant- and animal-sourced foods. Overall, differences between high- and low-UPF variants were modest, with broadly overlapping food group patterns (Supplementary Figure S3). As expected, higher adherence to the hPDI was associated with higher mNQI scores and higher adherence to the uPDI with lower scores, with minimal distinction between high- and low-UPF variants (Fig. 2).

### Ultra-processed plant-based diets, all-cause mortality and chronic disease

**Fig. 3:**
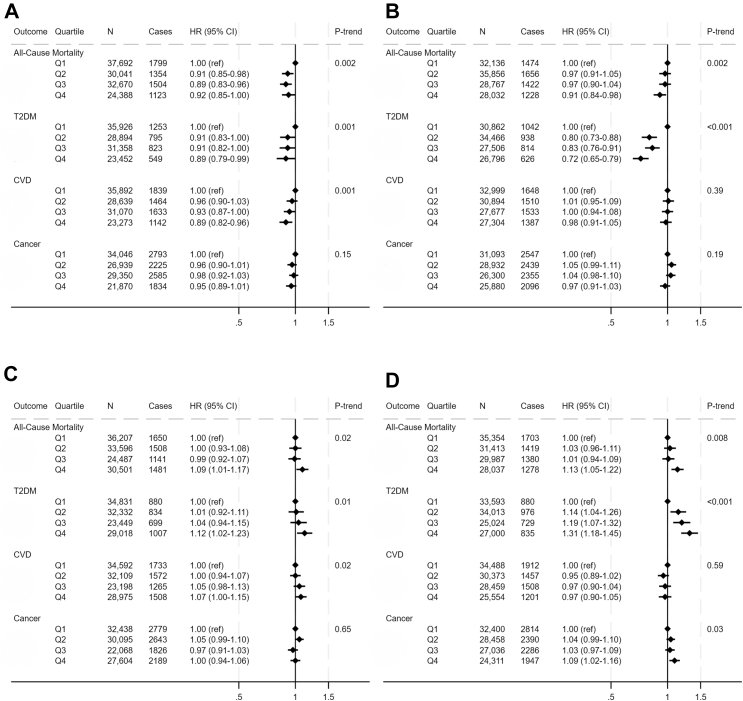
**Multivariable adjusted hazard ratios (95% confidence intervals) of all-cause mortality (n = 124,791), T2DM (n = 119,630), CVD (n = 118,874), and cancer (n = 112,205) across sex-specific quartiles (Q) of: (A) high-UPF hPDI, (B) low-UPF hPDI, (C) high-UPF uPDI, and (D) low-UPF uPDI.** All models adjusted for sex, education, ethnicity, BMI, physical activity, smoking status, alcohol intake, energy intake, multimorbidity index, polypharmacy, blood pressure medications, blood thinning medications, cholesterol lowering medications, Townsend deprivation index, and number of completed dietary assessments; stratified by age (5-year categories) and region. All-cause mortality models also adjusted for CVD and cancer at baseline; T2DM models also adjusted for CVD and cancer at baseline; CVD models also adjusted for T2DM and cancer at baseline; cancer models also adjusted for CVD and T2DM at baseline, menopausal status and menopause hormone treatment. For high-UPF analyses, models also adjusted intake of fruit, nuts, animal-fat, fruit juice and eggs. For low-UPF analyses, models also adjusted for intake of sugar-sweetened beverages, sweets and desserts and miscellaneous animal-based foods. P-trend is for linear trend. Abbreviations: UPF, ultra-processed food; hPDI, healthful plant-based diet index; uPDI, unhealthful plant-based diet index; BMI, Body Mass Index; HR, hazard ratio; CI, confidence interval; T2DM, type 2 diabetes mellitus; CVD, cardiovascular disease.

In fully adjusted models, greater adherence to healthful PBDs—whether characterised by a higher or lower contribution from UPFs—was associated with lower all-cause mortality [HR_Q4vsQ1_ (95% CI); high-UPF hPDI: 0.92 (0.85–1.00), *P*-trend = 0.002; low-UPF hPDI: 0.91 (0.84–0.98), *P*-trend = 0.002]. In contrast, greater adherence to unhealthful PBDs, regardless of processing level, was associated with higher all-cause mortality [high-UPF uPDI: 1.09 (1.01–1.17), *P*-trend = 0.02; low-UPF uPDI: 1.13 (1.05–1.22), *P*-trend = 0.008] (Fig. 3; Supplementary Table S11).

Similar patterns were observed for T2DM. Greater adherence to healthful PBDs, whether with higher or lower ultra-processed food content, was associated with lower T2DM risk [HR_Q4vsQ1_ (95% CI): high-UPF hPDI: 0.89 (0.79–0.99), *P*-trend = 0.001; low-UPF hPDI: 0.72 (0.65–0.79), *P*-trend <0.001]. In contrast, greater adherence to unhealthful PBDs was associated with higher T2DM risk across both processing strata [high-UPF uPDI: 1.12 (1.02–1.23), *P*-trend = 0.01; low-UPF uPDI: 1.31 (1.18–1.45), *P*-trend = <0.001] (Fig. 3; Supplementary Table S11).

For CVD, greater adherence to a healthful PBD with a higher contribution from UPFs was associated with lower CVD risk [HR_Q4vsQ1_ (95% CI): 0.89 (0.82–0.96), *P*-trend = 0.001], whereas no clear association was observed for the low-UPF healthful pattern. Conversely, greater adherence to an unhealthful PBD with a higher contribution from UPFs was associated with higher CVD risk [1.07 (1.00–1.15), *P*-trend = 0.02], with no corresponding association for the low-UPF unhealthful pattern (Fig. 3; Supplementary Table S11).

No significant associations were observed between either healthful PBD variant and cancer risk; however, greater adherence to an unhealthful PBD with lower UPF content was associated with higher cancer risk [HR_Q4vsQ1_ (95% CI): 1.09 (1.02–1.16), *P*-trend = 0.03] (Fig. 3; Supplementary Table S11).

Adjusted cumulative hazard plots for all-cause mortality, T2DM, CVD, and cancer are shown in Supplementary Figures S4–S7.

### Subgroup and sensitivity analyses

**Fig. 4:**
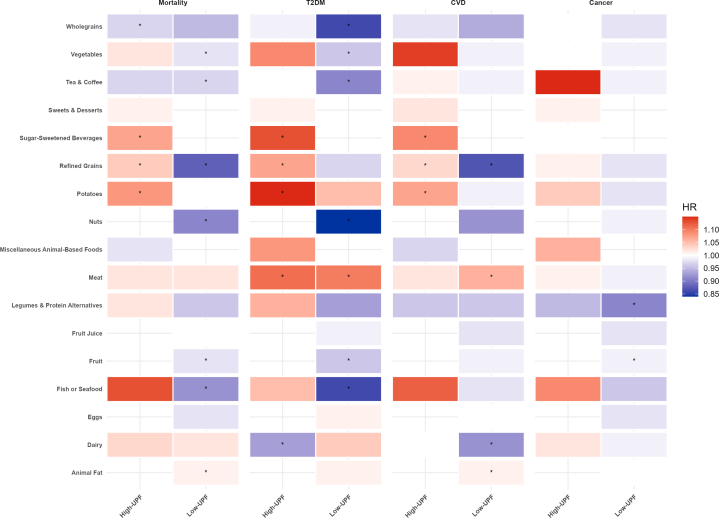
**Heatmap of multivariable adjusted hazard ratios for high-ultra-processed and low-ultra-processed PDI food groups in relation to all-cause mortality, T2DM, CVD, and cancer.** Colours indicate the direction and strength of associations: red denotes positive associations (higher risk), and dark purple denotes negative associations (lower risk); deeper shades reflect stronger associations. Statistical significance is indicated by: ∗p < 0.05. All models adjusted for sex, education, ethnicity, BMI, physical activity, smoking status, alcohol intake, energy intake, multimorbidity index, polypharmacy, blood pressure medications, blood thinning medications, cholesterol lowering medications, Townsend deprivation index, and number of completed dietary assessments; stratified by age (5-year categories) and region. All-cause mortality models also adjusted for CVD and cancer at baseline; T2DM models also adjusted for CVD and cancer at baseline; CVD models also adjusted for T2DM and cancer at baseline; cancer models also adjusted for CVD and T2DM at baseline, menopausal status and menopause hormone treatment. Abbreviations: UPF, ultra-processed food; PDI, plant-based diet index; hPDI, healthful plant-based diet index; uPDI, unhealthful plant-based diet index; BMI, Body Mass Index; HR, hazard ratio; CI, confidence interval; T2DM, type 2 diabetes mellitus; CVD, cardiovascular disease.

Higher intakes of fruit, vegetables, nuts, tea/coffee, and fish/seafood were associated with lower all-cause mortality and T2DM, while fruit, legumes, and vegetarian protein alternatives were linked to lower cancer incidence (Fig. 4; Supplementary Table S12). Wholegrain intake was inversely associated with T2DM risk when consumed in low-UPF forms and with all-cause mortality when contributing primarily from ultra-processed forms. Similarly, refined grains with lower ultra-processed content were associated with lower mortality and CVD risk. Dairy intake was inversely associated with CVD risk when consumed predominantly as low-UPF foods and with T2DM risk when contributing from ultra-processed sources.

In contrast, higher intakes of refined grains, potatoes (particularly when contributing from UPFs), and SSBs were associated with higher risks of mortality, T2DM, and CVD. Higher meat intake was associated with higher T2DM risk regardless of processing level and with higher CVD risk when consumed primarily as low-UPF foods, while higher animal fat intake was associated with higher CVD risk (Fig. 4; Supplementary Table S12). No significant associations were found for fruit juice, sweets/desserts, eggs, or miscellaneous animal-based foods (Fig. 4; Supplementary Table S12).

Leave-one-out analyses confirmed robustness, though some food groups influenced results. For the low-UPF hPDI, associations were stable; for the high-UPF hPDI, inverse associations with mortality, T2DM, and CVD attenuated when SSBs were excluded (Supplementary Table S13). The high-UPF uPDI was more sensitive—associations with mortality and CVD weakened after excluding wholegrains, potatoes, or SSBs, and with T2DM after removing refined grains, potatoes, SSBs, or dairy. The low-UPF uPDI remained largely robust, though the mortality association became borderline when fruit, vegetables, or nuts were excluded (Supplementary Table S14).

Adjustment for nutrient quality (mNQI) produced similar patterns with slightly attenuated estimates (Supplementary Table S15). Analyses using low-UPF:high-UPF PDI ratios showed no consistent associations with outcomes (Supplementary Table S16), aside from an isolated positive association with cancer in the highest quartile. No significant effect modification by overall UPF intake was observed for either hPDI or uPDI (Supplementary Table S17–S18). Sex-stratified analyses showed broadly consistent associations across men and women, with no evidence of effect modification by sex (all P-interaction >0.05) (Supplementary Table S19).

Additional sensitivity analyses restricted to all-cause mortality supported the robustness of the findings. Exclusion of deaths occurring within one year of the last dietary assessment yielded similar associations, with inverse relationships for the hPDI and positive associations for the uPDI remaining largely unchanged (Supplementary Table S20). Likewise, restricting follow-up to events occurring before 1 January 2020 to account for the potential influence of the COVID-19 pandemic produced consistent results (Supplementary Table S21), with no material differences in the magnitude or direction of associations.

## Discussion

In this large prospective study, higher adherence to healthy PBDs, whether high- or low-UPF, was associated with lower risks of all-cause mortality, T2DM, and CVD. Associations were slightly stronger for the low-UPF hPDI, suggesting additional benefits of diets rich in minimally processed, nutrient-dense plant foods. In contrast, unhealthy PBDs were consistently associated with higher risks regardless of UPF content, suggesting that poor diet quality may outweigh benefits of lower processing. Food group analyses highlighted the role of specific UPFs: SSBs appeared to drive adverse associations among unhealthful PBDs, whereas wholegrain UPFs were linked to lower risks.

Evidence on the intersection of PBDs and UPFs remains limited. Prior research suggests that vegetarians and vegans tend to consume more UPFs than meat eaters, largely due to plant-based substitutes,^16^ with similar findings from the UK Biobank^17^ and smaller studies showing that plant-based meat alternatives can contribute substantially to energy intake.^34^ Recent evidence from the Moli-sani cohort further indicates that the cardiometabolic benefits of high adherence to the Mediterranean diet are attenuated when UPF consumption is high,^35^ underscoring the importance of considering diet quality and UPF consumption jointly. The NutriNet-Santé cohort reported no cardiovascular benefit for high-UPF healthy PBDs,^36^ whereas we observed an inverse association between high-UPF hPDI and CVD. Differences across cohorts may reflect variation in dietary assessment, population characteristics, or outcome definitions. Our findings align with emerging evidence suggesting that both health and environmental impacts of PBDs may be driven more by the quality and proportion of plant foods than by processing level,^37^ suggesting that UPF-containing PBDs are not inherently harmful. This may reflect heterogeneity within UPFs, with detrimental effects concentrated in specific categories, such as SSBs, while nutrient-dense UPFs, such as fortified products or wholegrain cereals, may still contribute to a healthful dietary pattern.

Our findings are particularly relevant given the growing popularity of plant-based alternatives, which are often energy-dense, refined, and nutrient-poor. In this study, both high- and low-UPF hPDIs were associated with similarly high mNQI scores, reflecting greater fibre and lower saturated fat intakes, indicating that healthful PBDs can retain nutrient quality even with some UPFs. Differences between high- and low-UPF hPDIs were modest, with the former showing slightly higher calcium and lower cholesterol. In contrast, both uPDIs showed poorer nutrient profiles, including lower fibre, protein, calcium, and vitamin B12, particularly in low-UPF variants. These nutritional contrasts may explain why cardiometabolic associations were not uniformly stronger for low-UPF diets, suggesting that processing per se may be less important when the overall dietary pattern is healthful.

Modest differences between high- and low-UPF PDIs regarding associations with mortality and disease risks may reflect limitations of binary UPF categorisation. As seen in other cohorts,^37^ PBDs often include both minimally and highly processed foods, making it difficult to fully separate processing from nutrient quality. Nutrient quality may partially offset potential risks due to non-nutritional constituents of UPFs, such as additives (e.g., emulsifiers, sweeteners), packaging-derived contaminants (e.g., acrolein), or altered food matrices. These factors have been linked to inflammation, oxidative stress, impaired satiety signalling, and gut dysbiosis,^38^, ^39^, ^40^, ^41^ which may explain weaker associations for UPF-rich PBDs in some cases. However, our findings and recent analyses from other cohorts^42^ suggest that non-nutritive risks of UPFs may be negligible or restricted to a limited number of UPFs, compared to the effects of overall diet quality. Our food group analyses underline that some UPFs, particularly those rich in wholegrains, may be beneficial. Overall, our study strongly supports the notion that, while the consumption of individual UPFs such as SSBs may be harmful, UPF consumption per se is not, especially when the nutrient profile of the foods consumed is high.

Environmental sustainability is another important dimension in the context of UPFs. While PBDs generally have lower environmental footprints than animal-based diets,^4^ certain highly processed plant-based alternatives may entail greater industrial energy use, increased packaging and transport demands, and reliance on intensive crop systems that may undermine biodiversity and ecological resilience.^43^ In this context, the EAT-Lancet Commission report advises that sustainable diets should consist of minimally processed foods, while ultra-processed foods and beverages should be limited.^8^ However, recent modelling work from the EPIC cohort integrating food biodiversity, processing levels, and the EAT-Lancet framework demonstrated that achieving optimal nutrient adequacy and environmental sustainable requires balanced consideration of food diversity, nutrient density, and processing, rather than blanket exclusion of all UPFs.^44^ Similarly, emerging real-world evidence indicates that healthful PBDs can still confer environmental co-benefits even when they include some UPFs,^37^ aligning with our findings that select nutrient-dense UPFs may form part of a sustainable and health-promoting dietary pattern.

This study has several strengths, including its prospective design, large sample size, long follow-up (∼10 years), and robust case ascertainment. Key innovations include modifying PDIs by UPF classification and applying the mNQI to evaluate nutrient quality. However, several limitations should be considered. Firstly, the observational design precludes causal inference, and residual confounding remains possible despite multivariable adjustment; moreover, while we chose to consider BMI as a confounder, it may in part lie on the causal pathways between diet quality and disease risks. Dietary intake was self-reported using the Oxford WebQ which, despite validation,^21^ remains subject to measurement error and misclassification. Although analyses were restricted to participants with at least two valid 24-h dietary assessments, the majority of included participants completed only two recalls. As such, dietary exposures may not fully capture long-term habitual intake, particularly for episodically consumed foods. Nevertheless, previous studies indicate that the reliability of the PDI indices over time is good.^28^^,^^29^ Secondly, the UK Biobank has a notably low response rate to the initial recruitment invitation (approximately 5%), which is substantially lower than that reported in many population-based studies. As a result, participants are generally healthier, more educated, and health-conscious than the underlying population, limiting the external validity and generalisability of findings. In addition, a substantial proportion of the recruited cohort was excluded from the present analyses, primarily due to missing dietary data or insufficient repeated 24-h dietary assessments. Although these exclusions reflect the requirement for reliable estimation of habitual dietary intake rather than selective removal based on health status, they may have further accentuated selection bias, as participants with complete dietary data tend to have more favourable health profiles. Thirdly, temporal context should be considered when interpreting these findings. Dietary intake was assessed primarily between 2009 and 2012, a period when the availability, formulation, and market penetration of ultra-processed plant-based foods differed substantially from the contemporary food environment. Since then, the range and diversity of ultra-processed plant-based products—particularly meat and dairy alternatives—has expanded markedly, alongside reformulation efforts aimed at improving nutrient profiles. Consequently, these findings may not fully generalise to current consumption patterns. In addition, part of the follow-up period occurred after the introduction of the UK Soft Drinks Industry Levy in 2018, which led to widespread sugar reduction in beverages. Although dietary exposure in this study reflects pre-policy intake, future studies incorporating repeated dietary assessments are needed to evaluate how ongoing reformulation and policy interventions may modify the health effects of UPFs. Methodological considerations should also be noted. The Nova classification system may not fully capture all dimensions of processing relevant to health, and its application may obscure important nutritional distinctions within food categories. For example, many industrially produced breads, including wholegrain breads, are classified as ultra-processed despite evidence linking wholegrain consumption to reduced chronic disease and mortality risk.^45^ Additionally, the Oxford WebQ does not reliably distinguish between homemade and commercially prepared foods, meaning some items (e.g., desserts, coleslaw, pizza) may have been misclassified with respect to processing level. Construction of high- and low-UPF PDIs required exclusion of food groups without relevant items (e.g., fruit excluded from high-UPF indices; SSBs excluded from low-UPF indices), which may have influenced associations, although models accounted for these exclusions. Disease ascertainment relied on linked hospital and mortality records, potentially missing primary care-managed cases. Missing data were handled using indicator categories to retain participants in analyses. While this approach minimises data loss, it may introduce bias if missingness is not random; however, missingness was generally low across covariates. Finally, multiple testing was not applied given the primary focus on overall dietary patterns.

### Conclusion

Healthy PBDs are associated with lower risks of mortality, T2DM, and CVD, even when they include some UPFs, suggesting that diets centred on nutrient-dense plant foods remain beneficial despite the presence of processed products. In contrast, unhealthy PBDs—characterised by poorer nutrient profiles—were associated with adverse outcomes, regardless of UPF content, suggesting that overall dietary quality plays a central role in shaping health risk, beyond differences in food processing classification. While some nutrient-poor UPFs may attenuate the benefits of an otherwise healthful diet, the heterogeneity of UPFs means that some products with more favourable nutrient profiles (e.g., fortified or wholegrain-based products) may contribute positively when consumed as part of an overall balanced dietary pattern. Taken together, these findings support a more nuanced perspective in which health impacts of PBDs are shaped by the combined influence of diet quality, nutrient composition and processing, rather than processing level alone. From public health and sustainability standpoint, prioritising nutrient-rich, minimally processed plant-based foods remains essential, while acknowledging that select nutritionally favourable UPFs may play a pragmatic role in supporting accessible, realistic, and scalable dietary transitions.

## Contributors

Design and concept: AT, TK, AC; database development: AT; accessed and verified data: all authors; analysed and interpreted data: all authors; drafted manuscript: AT, TK, AC; provided critical review of the manuscript: all authors; supervised the data analysis: all authors; agreed to submit manuscript for publication: all authors; guarantors of the work: AT, TK, AC.

## Data sharing statement

UK Biobank data can be requested by all bona fide researchers for approved projects, including replication, through https://www.ukbiobank.ac.uk/. This research was conducted using UK Biobank funded and sourced data (application 64,426).

## Declaration of interests

None were reported.
