# Supplementary material for: Plant-Based Diets, Ultra-Processed Foods, and Risks of Mortality and Major Chronic Diseases: A Prospective Cohort Study
Source: Lancet Reg Health Eur. 2026 Jun 5;67:101736. doi: 10.1016/j.lanepe.2026.101736 (PMC13264357; doi:10.1016/j.lanepe.2026.101736)
Supplement: Supplementary Material [file mmc1.docx]

**Supplementary file 1 –Plant-Based Diets, Ultra-Processed Foods, and Risks of Mortality and Major Chronic Diseases: A Prospective Cohort Study**

***Thompson et al.***

**Contents**

[**Methods S1. Covariate Assessment** 3](#_Toc231211183)

[**Figure S1.** **Modified PDIs by Food Group and UPF Category** 4](#_Toc231211184)

[**Figure S2.** **Heatmap of Pearson correlations between PDIs by UPF level (n=124,791)** 5](#_Toc231211185)

[**Figure S3.** **Radar plots of food group intakes across quartiles (Q1-Q4) of plant-based diet indices (hPDI and uPDI) stratified by UPF consumption** 6](#_Toc231211186)

[**Figure S4. Adjusted cumulative hazard of all-cause mortality by quartiles of high- and low-UPF hPDI and uPDI** **(attained age <80 years)** 7](#_Toc231211187)

[**Figure S5. Adjusted cumulative hazard of T2DM by quartiles of high- and low-UPF hPDI and uPDI (attained age <80 years)** 8](#_Toc231211188)

[**Figure S6. Adjusted cumulative hazard of CVD by quartiles of high- and low-UPF hPDI and uPDI (attained age <80 years)** 9](#_Toc231211189)

[**Figure S7. Adjusted cumulative hazard of cancer by quartiles of high- and low-UPF hPDI and uPDI (attained age <80 years)** 10](#_Toc231211190)

[**Table S1. Classification of Oxford WebQ food items into PDI food groups by Nova processing level** 11](#_Toc231211191)

[**Table S2. Follow-up time across study outcomes** 14](#_Toc231211192)

[**Table S3.** **Covariate coding and categorisation information** 15](#_Toc231211193)

[**Table S4.** **Comparison of baseline characteristics of the UK Biobank, included (n=124,836) and excluded (n=291,306) participants** 18](#_Toc231211194)

[**Table S5**. **Key nutrient intakes across quartiles (Q) of high-ultra-processed hPDI (n=124,791)** 19](#_Toc231211195)

[**Table S6**. **Key nutrient intakes across quartiles (Q) of low-ultra-processed hPDI (n=124,791)** 20](#_Toc231211196)

[**Table S7**. **Key nutrient intakes across quartiles (Q) of high-ultra-processed uPDI (n=124,791)** 21](#_Toc231211197)

[**Table S8**. **Key nutrient intakes across quartiles (Q) of low-ultra-processed uPDI (n=124,791)** 22](#_Toc231211198)

[**Table S9**. **Key nutrient intakes across quartiles (Q) of the ratio of low-UPF: high-UPF hPDI (n=124,791)** 23](#_Toc231211199)

[**Table S10**. **Key nutrient intakes across quartiles (Q) of the ratio of low-UPF: high-UPF uPDI (n=124,791)** 24](#_Toc231211200)

[**Table S11.** **Hazard ratios (95% confidence intervals) of mortality and major chronic diseases across sex-specific quartiles (Q) of high-ultra-processed and low-ultra-processed hPDI and uPDI** 25](#_Toc231211201)

[**Table S12.** **Hazard ratios (95% confidence intervals) of all-cause mortality (n=124,791), T2DM (n=119,630), CVD (n=118,874), and cancer (n=112,205) across high-ultra-processed and low-ultra-processed PDI food groups** 27](#_Toc231211202)

[**Table S13.** **Hazard ratios (95% confidence intervals) of all-cause mortality (n=124,791), T2DM (n=119,630), CVD (n=118,874), and cancer (n=112,205) across high-ultra-processed and low-ultra-processed hPDI (10-point increments), systematically excluding each food group** 30](#_Toc231211203)

[**Table S14.** **Hazard ratios (95% confidence intervals) of all-cause mortality (n=124,791), T2DM (n=119,630), CVD (n=118,874), and cancer (n=112,205) across high-ultra-processed and low-ultra-processed uPDI (10-point increments), systematically excluding each food group** 33](#_Toc231211204)

[**Table S15.** **Sensitivity analysis showing hazard ratios (95% confidence intervals) of all-cause mortality (n=124,791), T2DM (n=119,630), CVD (n=118,874), and cancer (n=112,205) across sex-specific quartiles (Q) of high-ultra-processed and low-ultra-processed hPDI and uPDI, further adjusting for a Modified Nutrient Quality Index** 36](#_Toc231211205)

[**Table S16.** **Hazard ratios (95% confidence intervals) of all-cause mortality (n=124,791), T2DM (n=119,630), CVD (n=118,874), and cancer (n=112,205) across quartiles (Q) of a ratio of low-ultra-processed: high-ultra-processed hPDI and uPDI** 38](#_Toc231211206)

[**Table S17.**  **Hazard ratios (95% confidence intervals) of all-cause mortality (n=124,791), T2DM (n=119,630), CVD (n=118,874), and cancer (n=112,205) across hPDI (10-point increments), stratified by UPF intake** 39](#_Toc231211207)

[**Table S18.**  **Hazard ratios (95% confidence intervals) of all-cause mortality (n=124,791), T2DM (n=119,630), CVD (n=118,874), and cancer (n=112,205) across uPDI (10-point increments), stratified by UPF intake** 40](#_Toc231211208)

[**Table S19. Sensitivity analysis showing hazard ratios (95% confidence intervals) of all-cause mortality (n=124,791), T2DM (n=119,630), CVD (n=118,874), and cancer (n=112,205) across high-ultra-processed and low-ultra-processed hPDI and uPDI (10-point increments), stratified by Sex** 41](#_Toc231211209)

[**Table S20. Hazard ratios (95% confidence intervals) of mortality across sex-specific quartiles (Q) of high-ultra-processed and low-ultra-processed hPDI and uPDI, excluding deaths occurring within 1 year of the last dietary assessment** 43](#_Toc231211210)

[**Table S21. Hazard ratios (95% confidence intervals) of mortality across sex-specific quartiles (Q) of high-ultra-processed and low-ultra-processed hPDI and uPDI, with follow-up restricted to before 2020 (pre-COVID-19 pandemic)** 44](#_Toc231211211)

**Methods S1. Covariate Assessment**

Between 2006 and 2010, participants attended a UK Biobank assessment centre where they completed a comprehensive baseline assessment. Data on sociodemographic characteristics (e.g., age, sex, ethnicity, education, and area-level deprivation), lifestyle factors (e.g., smoking and physical activity), and medical history (including female-specific factors) were collected via touchscreen questionnaire and/or verbal interview. Physical activity was assessed using the short-form International Physical Activity Questionnaire, and body mass index (BMI) was calculated from height and weight measured by trained staff. Multimorbidity and polypharmacy were derived from self-reported data obtained during verbal interview, while use of specific medications (e.g., cholesterol-lowering, blood pressure-lowering, and blood-thinning agents) was captured via touchscreen questionnaire. Prevalent disease was determined by combining self-reported information with linked hospital records. Area-level socioeconomic status was estimated using the Townsend deprivation index. Dietary intake was assessed using the self-administered Oxford WebQ 24-hour dietary questionnaire.


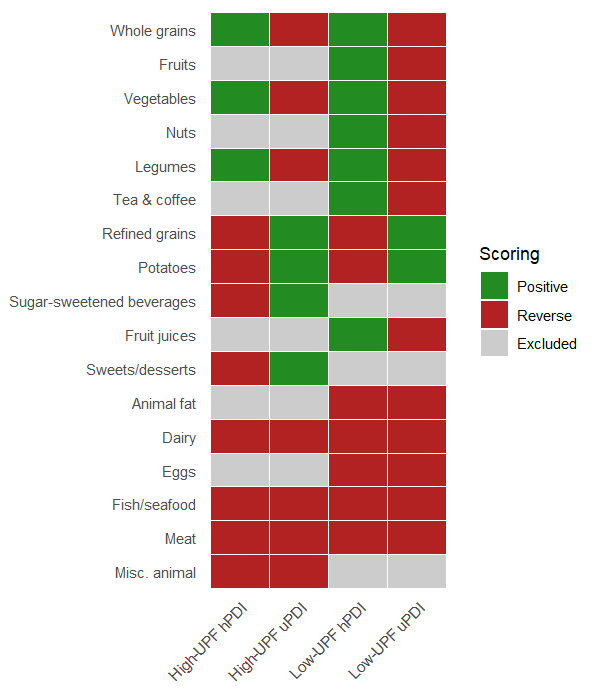


**Figure S1.** **Modified PDIs by Food Group and UPF Category**

*Abbreviations: UPF, ultra-processed food; hPDI, healthful plant-based diet index; uPDI, unhealthful plant-based diet index.*

*
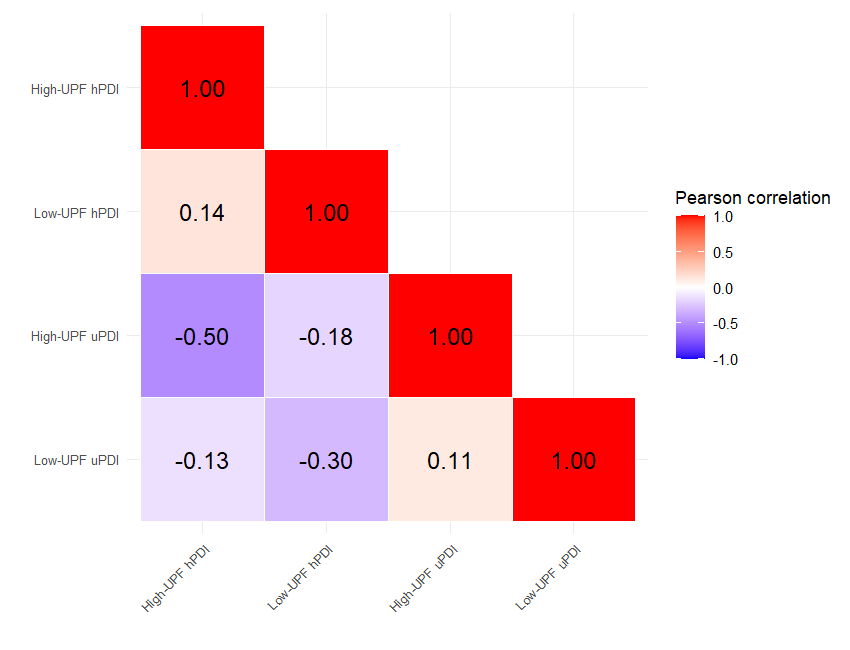
*

**Figure S2.** **Heatmap of Pearson correlations between PDIs by UPF level (n=124,791)**

All correlations are statistically significant (*P* <0.001).

*Abbreviations: UPF, ultra-processed food; hPDI, healthful plant-based diet index; uPDI, unhealthful plant-based diet index.*


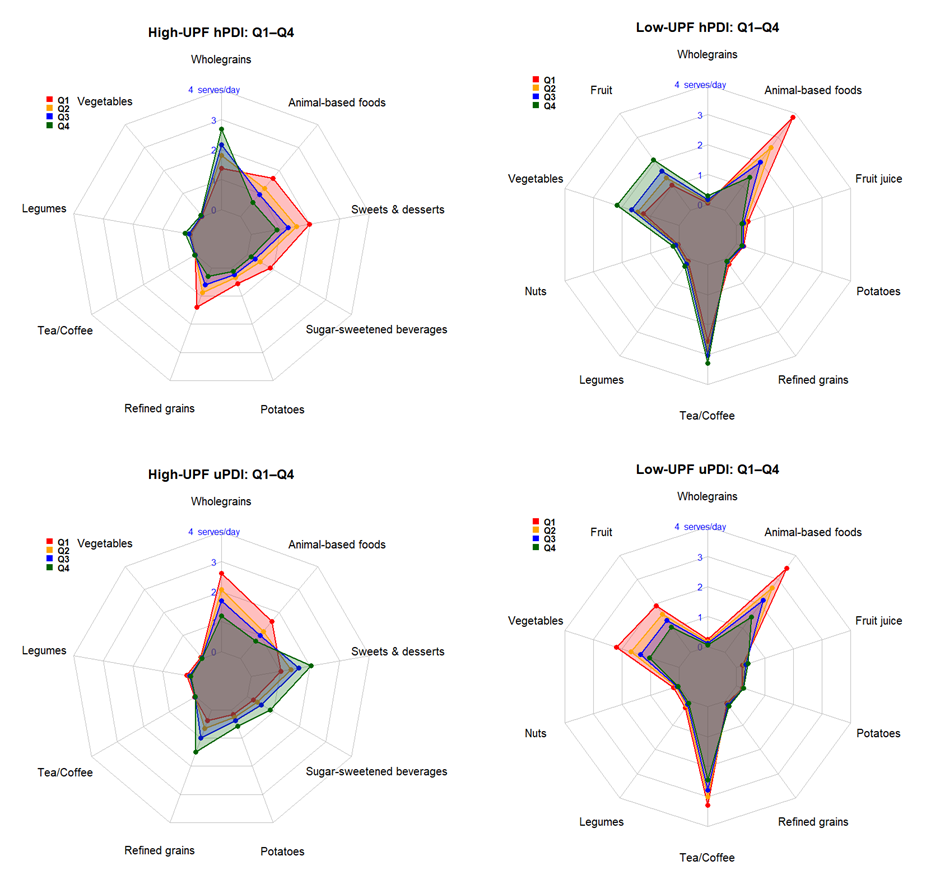


**Figure S3.** **Radar plots of food group intakes across quartiles (Q1-Q4) of plant-based diet indices (hPDI and uPDI) stratified by UPF consumption**

*Note: "Animal-based foods" includes dairy, fish, meat, and miscellaneous for UPF scores, and animal fat, dairy, eggs, fish, and meat for non-UPF scores.*

*Abbreviations: Q, quartile; UPF, ultra-processed food; hPDI, healthful plant-based diet index; uPDI, unhealthful plant-based diet index*

**Figure S4. Adjusted cumulative hazard of all-cause mortality by quartiles of high- and low-UPF hPDI and uPDI** **(attained age <80 years)**

*Abbreviations: Q, quartile; UPF, ultra-processed food; hPDI, healthful plant-based diet index; uPDI, unhealthful plant-based diet index.*

**Figure S5. Adjusted cumulative hazard of T2DM by quartiles of high- and low-UPF hPDI and uPDI (attained age <80 years)**

*Abbreviations: Q, quartile; UPF, ultra-processed food; hPDI, healthful plant-based diet index; uPDI, unhealthful plant-based diet index; T2DM, type 2 diabetes mellitus.*

**Figure S6. Adjusted cumulative hazard of CVD by quartiles of high- and low-UPF hPDI and uPDI (attained age <80 years)**

*Abbreviations: Q, quartile; UPF, ultra-processed food; hPDI, healthful plant-based diet index; uPDI, unhealthful plant-based diet index; CVD, cardiovascular disease.*

**Figure S7. Adjusted cumulative hazard of cancer by quartiles of high- and low-UPF hPDI and uPDI (attained age <80 years)**

*Abbreviations: Q, quartile; UPF, ultra-processed food; hPDI, healthful plant-based diet index; uPDI, unhealthful plant-based diet index.*

| **Table S1. Classification of Oxford WebQ food items into PDI food groups by Nova processing level** | | | |
| --- | --- | --- | --- |
| **Food groups** | **High-UPF Items (Nova 4)** | **Low-UPF Items (Nova 1-3)** | **Scoring (hPDI/uPDI)** |
| **Healthy Plant Food** |  |  |  |
| Whole grains | **Muesli, oat crunch, bran cereal,** **cereal bar, non-white bread (flour types, brown, wholemeal, other type), seeded or other bread, crispbread, whole-wheat cereal, other cereal** | Porridge, whole meal pasta, brown rice, couscous, other cooked grains (such as bulgur). | Positive / Reverse |
| Fruits |  | Mixed fruit, apple, banana, berries, cherries, grapefruit, grapes, mango, melon, orange, orange-like small fruits, peach/nectarine, pear, pineapple, plum, other fruits, stewed/cooked fruit, prunes, other dried fruit | Positive / Reverse |
| Vegetables | **Coleslaw** | Mixed vegetables, vegetable pieces, side salad, beetroot, broccoli, butternut squash, cabbage/kale, carrots, cauliflower, celery, courgette, cucumber, garlic, leeks, lettuce, mushrooms, onion, parsnip, sweet peppers, spinach, sprouts, sweetcorn, sweet potato, fresh tomatoes, cooked or tinned tomatoes, turnip/swede, watercress, other vegetable intake | Positive / Reverse |
| Nuts |  | Salted peanuts, unsalted peanuts, salted nuts, unsalted nuts, seeds | Positive / Reverse |
| Legumes, Vegetarian protein alternatives | **Beans (baked beans)**, **vegetarian sausages/burgers, tofu**, **Quorn, other vegetarian alternative** | Other beans or lentils, broad beans, green beans, peas, soy or vegetable milk | Positive / Reverse |
| Tea and coffee | **Other coffee drinks** | Instant coffee, filtered coffee, cappuccino, latte, espresso, standard tea, rooibos tea, green tea, herbal tea, other tea | Positive / Reverse |
| **Less Healthy Plant Food** |  |  |  |
| Refined grains | **Sweetened cereal, plain cereal, white bread, naan bread, garlic bread**, **Pancake, scotch pancake**, **croissant, scone**, **savoury or cheesy biscuits, other savoury snack, snackpot** | White pasta, white rice | Reverse / Positive |
| Potatoes | **Fried potatoes, crisps (e.g., potato chips)** | Boiled/baked potatoes, mashed potatoes | Reverse / Positive |
| Sugar-sweetened beverages | **Low calorie or diet drinks (e.g. fizzy, squash), carbonated (fizzy) drinks**, **squash or cordial** |  | Reverse / Positive |
| Fruit juices |  | Fruit smoothie, orange juice, grapefruit juice, other fruit/vegetable juice | Reverse / Positive |
| Sweets and desserts | **Double crust pie, single crust pie/flan, crumble topping, Yorkshire pudding, Danish pastry, fruitcake, cake, doughnuts, sponge pudding, other dessert, chocolate bar, white chocolate, milk chocolate, dark chocolate, chocolate-covered raisin, chocolate sweet, diet sweets, chocolate-covered biscuits, chocolate biscuits, sweet biscuits, other sweets** |  | Reverse / Positive |
| **Animal-derived Food** |  |  |  |
| Animal fat |  | Butter on bread/crackers (spreadable, low fat, normal fat, or unknown type), dairy spread on bread/crackers (very low fat, low fat, normal fat, unknown type) | Reverse / Reverse |
| Dairy | **Ice-cream, dairy smoothie, flavoured milk, yogurt, low fat cheese spread, cheese spread, milk-based pudding, other milk-based pudding, cheesecake** | Milk, low fat hard cheese, hard cheese, soft cheese, blue cheese, cottage cheese, feta cheese, mozzarella cheese, goat's cheese, other cheese | Reverse / Reverse |
| Eggs |  | Scotch egg, whole eggs, omelettes or scrambled egg, eggs in sandwiches, other egg dishes | Reverse / Reverse |
| Fish or seafood | **Breaded fish, battered fish** | Tinned tuna, oily fish, white fish, prawns, lobster/crab, shellfish, other fish intake | Reverse / Reverse |
| Meat | **Sausage, Crumbed or deep-fried poultry, ham, liver** | Beef, pork, lamb, poultry, bacon, other meat intake | Reverse / Reverse |
| Miscellaneous animal-based foods | **Pizza, Indian snacks** |  | Reverse / Reverse |
| Food items in **bold** are ultra-processed plant foods.  Positive scoring indicates that higher intake of a food group contributes to a higher index score, while reverse scoring indicates that higher intake contributes to a lower index score. For positively scored food groups, non-consumers receive a score of 1, and remaining participants are ranked into sex-specific quartiles and assigned scores from 2 (lowest intake) to 5 (highest intake). For reverse-scored food groups, this scoring is inverted.  High-UPF PDI variants include only Nova 4 foods within each food group; all other foods are excluded.  Low-UPF PDI variants include only Nova 1–3 foods within each food group; Nova 4 foods are excluded.  Some food groups contain no items in a given UPF category (e.g., fruits contain no high-UPF items, sugar-sweetened beverages contain no low-UPF items); these groups are excluded from the respective index variant.  *Abbreviations: UPF, ultra-processed food; hPDI, healthful plant-based diet index; uPDI, unhealthful plant-based diet index.* | | | |

| **Table S2. Follow-up time across study outcomes** | | | | | | |
| --- | --- | --- | --- | --- | --- | --- |
| **Outcome** | **N (analytic sample)** | **Events (n)** | **Mean follow-up, years (SD)** | **Median (IQR), years** | **Range, years** | **Person-years** |
| All-cause mortality | 124,791 | 5,780 | 10·5 (1·3) | 10·6 (0·5) | 0·0-11·8 | 1,307,953 |
| T2DM | 119,630 | 3,420 | 10·3 (1·5) | 10·5 (0.5) | 0·0-11·7 | 1,229,744 |
| CVD | 118,874 | 6,078 | 10·2 (1·7) | 10·5 (0·5) | 0·0-11·7 | 1,211,854 |
| Cancer | 112,205 | 9,437 | 8·3 (1·7) | 8·7 (0·5) | 0·0-10·4 | 934,580 |
| *Abbreviations: SD, standard deviation; IQR, interquartile range; T2DM, type 2 diabetes mellitus; CVD, cardiovascular disease.* | | | | | | |

| **Table S3.** **Covariate coding and categorisation information** | | |
| --- | --- | --- |
| **Variables** | **Categorisation** | **UK Biobank variable description and data-field ID** |
| Demographics |  |  |
| Age | Age (years): 5-year categories (<45 years, 45–, 50–, 55-, 60–, ≥65 years) | Age at recruitment (ID: 21022)^a^ |
| Sex | Female; Male | Sex (ID: 31)^a^ |
| Ethnicity | Asian, Black, Mixed, White, Other; Unknown/Missing (0.3%) | Ethnic background (ID: 21000)^a^ |
| Region | London; Wales; North-West England; North-East England; Yorkshire; West Midlands; East Midlands; South-East England; South-West England; Scotland | UK Biobank assessment centre (ID:54)^a^ |
| Socioeconomic status | |  |
| Education | Low: CSEs or equivalent, O levels/GCSEs or equivalent; Medium: A levels/AS levels or equivalent, NVQ or HND or HNC or equivalent; High: College or University degree, other professional qualifications e.g. nursing, teaching; Unknown/Missing (6.8%) | Qualifications (ID: 6138)^a^ |
| Townsend deprivation index | Quintiles ranging from least deprived (quintile 1) to most deprived (quintile 5); Unknown/ Missing (0.1%) | Townsend deprivation index (ID: 189)^a^ |
| Lifestyle and Environment | |  |
| Alcohol intake | Alcohol intake (g/day) (continuous scale) | Alcohol (ID: 26030)^b^ |
| Smoking status | Never; Previous; Current; Unknown/Missing (0.2%) | Smoking status (ID: 20116)^a^ |
| Physical activity | METs hr/week quintiles ranging from least active (quintile 1) to most active (quintile 5); Unknown/Missing (1.8%) | Duration of walks (ID: 874)^a^; Number of days/week walked 10+ minutes (ID: 864) ^a^; Duration of moderate activity (ID: 894)^a^; Number of days/week of moderate physical activity 10+ minutes (ID: 884)^a^; Duration of vigorous activity (ID: 914)^a^; Number of days/week of vigorous physical activity 10+ minutes (ID: 904)^a^ |
| Energy intake | Energy intake (kJ/day), calculated as the mean across 2-5 dietary assessments (continuous scale) | Energy (ID: 26002)^b^ |
| Number of completed dietary assessments | Number of dietary assessments completed (ranging from 2-5) | Number of diet questionnaires completed (ID: 20077)^b^ |
| Modified nutrient quality index | Derived by summing quartile-based scores for fibre, protein (positive scoring) and sugars, saturated fat, and sodium (reverse scoring); higher scores indicate better nutrient quality (continuous scale) | Englyst fibre (ID: 26017)^b^; protein (ID: 26005)^b^; saturated fat (ID:26014)^b^; total sugars (ID: 26011)^b^; sodium (ID: 26052)^b^ |
| Health status |  |  |
| BMI | Underweight (<18·5 kg/m^2^); Healthy weight (18·5-24·99 kg/m^2^); Overweight (25-29·99 kg/m^2^); Obese (≥30 kg/m^2^); Unknown/Missing (0.2%) | BMI (ID: 21001)^c^ |
| Multimorbidity | Number of pre-existing long-term conditions (0, 1, 2, ≥3) | Non-cancer illness code, self-reported (ID: 20002)^e^; Cancer diagnosed by doctor (ID: 2453)^a^ |
| Polypharmacy | Total number of self-reported medications taken at baseline (0, 1-3, 4-6, 7-9, ≥10); Unknown/Missing (0.1%) | Number of treatments/medications taken (ID: 137)^e^ |
| CVD at baseline | No; Yes | Diagnoses – ICD9 (ID:41271)^d^ / ICD10 (ID: 41270)^d^; Date of first in-patient diagnosis – ICD9 (ID: 41281)^d^ / ICD10 (ID: 41280)^d^; Non-cancer illness code, self-reported (ID: 20002)^e^ |
| Type 2 diabetes at baseline | No; Yes | Diagnoses – ICD9 (ID:41271)^d^ / ICD10 (ID: 41270)^d^; Date of first in-patient diagnosis – ICD9 (ID: 41281)^d^ / ICD10 (ID: 41280)^d^; Non-cancer illness code, self-reported (ID: 20002)^e^; Medication for cholesterol, blood pressure or diabetes (ID: 6177)^a^; Medication for cholesterol, blood pressure, diabetes, or take exogenous hormones (ID: 6153)^a^; Treatment/medication code (ID: 20003)^e^ ; Diabetes diagnosed by doctor (ID: 2443)^a^ |
| Cancer at baseline | No; Yes | Diagnoses – ICD9 (ID:41271)^d^ / ICD10 (ID: 41270)^d^; Date of first in-patient diagnosis – ICD9 (ID: 41281)^d^ / ICD10 (ID: 41280)^d^; Cancer code, self-reported (ID: 20001)^e^ |
| Menopausal status | No; Yes; Not sure (hysterectomy/other reason); Men; Unknown/Missing (0.1%) | Had menopause (women only) (ID: 2724)^a^ |
| HRT use | No; Yes; Unknown/Missing (0.1%) | Ever used hormone-replacement therapy (HRT) (ID: 2814)^a^ |
| Blood pressure medication | No; Yes; Unknown/Missing (10.9%) | Medication for cholesterol, blood pressure or diabetes (ID: 6177)^a^; Medication for cholesterol, blood pressure, diabetes, or take exogenous hormones (ID: 6153)^a^; Treatment/medication code (ID: 20003)^e^ |
| Blood thinning medication | No; Yes; Unknown/Missing (27.7%) | Medication for pain relief, constipation, heartburn (ID: 6154)^a^; Treatment/medication code (ID: 20003)^e^ |
| Cholesterol lowering medication | No; Yes; Unknown/Missing (14.2%) | Medication for cholesterol, blood pressure or diabetes (ID: 6177)^a^; Medication for cholesterol, blood pressure, diabetes, or take exogenous hormones (ID: 6153)^a^; Treatment/medication code (ID: 20003)^e^ |
| ^a^ Data collected at recruitment via touchscreen questionnaire (initial assessment visit (2006-2010). ^b^ Data collected from 24-hr online Oxford WebQ dietary questionnaire (assessment centre (April 2009 to September 2010; on-line cycle 1 (February 2011 to April 2011); on-line cycle 2 (June 2011 to September 2011); on-line cycle 3 (October 2011 to December 2011); on-line cycle 4 (April 2012 to June 2012). ^c^ Physical measurements (initial assessment visit (2006-2010). ^d^ Hospital inpatient admission data. ^e^ Data collected via verbal interview (initial assessment visit (2006-2010). *Abbreviations: CSEs, Certificate of Secondary Education; O levels, Ordinary levels; GCSEs, General Certificate of Secondary Education; A levels, Advanced levels; AS levels, Advanced Subsidiary levels; NVQ, National Vocational Qualification; HND, Higher National Diploma; HNC, Higher National Certificate; BMI, body mass index; MET, metabolic equivalent task; CVD, cardiovascular disease; HRT, hormone replacement therapy* | | |

| **Table S4.** **Comparison of baseline characteristics of the UK Biobank, included (n=124,836) and excluded (n=291,306) participants** | | | |
| --- | --- | --- | --- |
| **Characteristic** | **Whole cohort**  **(n=502,158)** | **Excluded participants**  **No dietary data**  **(n=291,306)** | **Included participants**  **Eligible dietary data available (≥2 dietary assessments)**  **(n=124,836)** |
| **Sex-Female** | 273,172 (54·4) | 157,021 (53·9) | 69,690 (55·8) |
| **Age at recruitment (years)** | 56·5 (8·1) | 56·9 (8·2) | 56·2 (7·8) |
| **BMI (kg/m^2^)** | 27·4 (4·8) | 27·8 (4·9) | 26·7 (4·6) |
| **BMI (kg/m^2^)** |  |  |  |
| **Underweight (<18.5)** | 2,625 (0·5) | 1,481 (0·5) | 719 (0·6) |
| **Healthy weight (18.5 to <25)** | 162,272 (32·3) | 84,963 (29·2) | 48,705 (39·0) |
| **Overweight (25 to <30)** | 211,978 (42·2) | 124,690 (42·8) | 50,842 (40·7) |
| **Obese (≥30)** | 122,179 (24·3) | 77,662 (26·7) | 24,288 (19·5) |
| **Ethnicity** |  |  |  |
| White | 472,388 (94·1) | 271,244 (93·1) | 120,602 (96·6) |
| Mixed | 2,950 (0·6) | 1,699 (0·6) | 667 (0·5) |
| Asian | 11,444 (2·3) | 7,908 (2·7) | 1,463 (1·2) |
| Black | 8,048 (1·6) | 5,436 (1·9) | 965 (0·8) |
| Other^a^ | 2,775 (0·6) | 3,015 (1·0) | 723 (0·6) |
| **Education level^b^** |  |  |  |
| Low | 83,248 (16·6) | 51,461 (17·7) | 17,126 (13·7) |
| Medium | 90,160 (18·0) | 52,472 (18·0) | 21,177 (17·0) |
| High | 233,394 (46·5) | 111,136 (38·2) | 78,040 (62·5) |
| **Smoking status** |  |  |  |
| Never | 273,342 (54·4) | 154,400 (53·0) | 71,333 (57·1) |
| Previous | 172,931 (34·4) | 98,122 (33·7) | 44,652 (35·8) |
| Current | 52,937 (10·5) | 36,402 (12·5) | 8,584 (6·9) |
| **Physical activity (MET-h/wk)** | 34·8 (48·8) | 36·5 (53·5) | 31·1 (38·5) |
| ^a^Other includes any race or ethnic group not otherwise specified.  ^b^Education is categorised as Low: CSEs or equivalent, O levels/GCSEs or equivalent; Medium: A levels/AS levels or equivalent, NVQ or HND or HNC or equivalent; High: College or University degree, other professional qualifications eg: nursing, teaching.  Data are expressed as mean (SD) or *n* (%), unless otherwise stated. Relative frequencies (%) include missing values which may not equate to 100%.  *Abbreviations: Q, quartile; BMI, body mass index; MET, metabolic equivalent of task; CSEs, Certificate of Secondary Education; O levels, Ordinary levels; GCSEs, General Certificate of Secondary Education; A levels, Advanced levels; AS levels, Advanced Subsidiary levels; NVQ, National Vocational Qualification; HND, Higher National Diploma; HNC, Higher National Certificate.* | | | |

| **Table S5**. **Key nutrient intakes across quartiles (Q) of high-ultra-processed hPDI (n=124,791)** | | | | | |
| --- | --- | --- | --- | --- | --- |
|  | **High-UPF hPDI** | | | | **Whole Sample** |
|  | **Mean (SD)** | | | | **Mean (SD)** |
| **Key nutrient intakes** | **Q1**  **(n=37,692)** | **Q2**  **(n=30,041)** | **Q3**  **(n=32,670)** | **Q4**  **(n=24,388)** | **(n=124,791)** |
| Modified Nutrient Quality Index | 11·5 (2·0) | 12·4 (2·0) | 12·9 (2·0) | 13·6 (1·9) | 12·5 (2·1) |
| Energy, kJ/day | 9158·3 (1955·1) | 8539·7 (1898·6) | 8292·7 (1900·7) | 7942·9 (1849·9) | 8545·2 (1959·1) |
| Calcium, mg/day | 1022·3 (289·9) | 972·2 (281·8) | 953·0 (284·0) | 929·7 (284·3) | 974·0 (287·4) |
| Protein, g/day | 82·8 (19·5) | 80·2 (19·1) | 79·1 (19·4) | 76·8 (19·9) | 80·0 (19·6) |
| Fibre, g/day | 16·7 (5·0) | 17·3 (5·4) | 18·1 (5·7) | 19·8 (6·4) | 17·8 (5·7) |
| Total fat, g/day | 79·5 (22·8) | 72·5 (22·3) | 69·4 (22·4) | 65·9 (22·6) | 72·5 (23·1) |
| Saturated fat, g/day | 30·1 (10·0) | 27·1 (9·6) | 25·6 (9·5) | 23·0 (9·2) | 26·8 (10·0) |
| Cholesterol, mg/day | 187·1 (92·2) | 182·4 (94·0) | 177·3 (95·6) | 165·9 (101·6) | 179·3 (95·7) |
| Iodine, µg/day | 219·8 (85·0) | 210·6 (81·6) | 205·3 (81·1) | 194·2 (82·6) | 208·8 (83·2) |
| Vitamin B12, µg/day | 6·2 (2·7) | 6·2 (2·7) | 6·2 (2·7) | 5·9 (2·8) | 6·1 (2·7) |
| Vitamin D, µg/day | 3·5 (2·1) | 3·6 (2·3) | 3·7 (2·5) | 3·8 (2·8) | 3·6 (2·4) |
| *Quartiles (Q) are based on index scores, where Q1 represents the lowest adherence and Q4 represents the highest adherence to each dietary index.*  *Recommended nutrient intakes for adults are provided for reference (UK Reference Nutrient Intakes/Dietary Reference Values): Energy ~8400 kJ/day (women) / ~10,500 kJ/day (men); Calcium 700 mg/day; Protein 45–55 g/day; Fibre 30 g/day; Total fat 70 g/day; Saturated fat <20 g/day; Cholesterol <300 mg/day; Iodine 140 µg/day; Vitamin B12 1.5 µg/day; Vitamin D 10 µg/day. Values are population-level references; individual requirements vary by sex, age, and health status.*  *Abbreviations: Q, quartile; UPF, ultra-processed food; hPDI, healthful plant-based diet index; SD, standard deviation.* | | | | | |

| **Table S6**. **Key nutrient intakes across quartiles (Q) of low-ultra-processed hPDI (n=124,791)** | | | | | | |
| --- | --- | --- | --- | --- | --- | --- |
|  | **Low-UPF hPDI** | | | | **Whole Sample** | |
|  | **Mean (SD)** | | | | **Mean (SD)** | |
| **Key nutrient intakes** | **Q1**  **(n=32,136)** | **Q2**  **(n=35,856)** | **Q3**  **(n=28,767)** | **Q4**  **(n=28,032)** | | **(n=124,791)** |
| Modified Nutrient Quality Index | 11·8 (2·0) | 12·3 (2·0) | 12·7 (2·0) | 13·3 (2·0) | | 12·5 (2·1) |
| Energy, kJ/day | 8921·4 (1940·5) | 8510·9 (1930·8) | 8432·2 (1963·6) | 8273·9 (1947·5) | | 8545·2 (1959·1) |
| Calcium, mg/day | 963·7 (282·8) | 958·3 (285·1) | 974·3 (287·0) | 1005·6 (293·5) | | 974·0 (287·4) |
| Protein, g/day | 85·0 (19·7) | 79·8 (19·1) | 78·5 (19·3) | 76·3 (19·0) | | 80·0 (19·6) |
| Fibre, g/day | 15·6 (4·8) | 16·9 (5·1) | 18·3 (5·4) | 20·9 (6·1) | | 17·8 (5·7) |
| Total fat, g/day | 78·6 (23·1) | 72·3 (22·5) | 70·3 (22·8) | 68·0 (22·9) | | 72·5 (23·1) |
| Saturated fat, g/day | 30·6 (10·3) | 27·2 (9·6) | 25·7 (9·4) | 23·3 (8·9) | | 26·8 (10·0) |
| Cholesterol, mg/day | 228·0 (102·7) | 183·4 (90·1) | 163·1 (84·9) | 134·7 (76·9) | | 179·3 (95·7) |
| Iodine, µg/day | 211·4 (83·4) | 207·2 (82·4) | 208·9 (83·2) | 207·6 (83·9) | | 208·8 (83·2) |
| Vitamin B12, µg/day | 6·7 (2·8) | 6·2 (2·7) | 6·0 (2·6) | 5·7 (2·6) | | 6·1 (2·7) |
| Vitamin D, µg/day | 4·2 (2·5) | 3·7 (2·3) | 3·4 (2·3) | 3·2 (2·3) | | 3·6 (2·4) |
| *Quartiles (Q) are based on index scores, where Q1 represents the lowest adherence and Q4 represents the highest adherence to each dietary index.*  *Recommended nutrient intakes for adults are provided for reference (UK Reference Nutrient Intakes/Dietary Reference Values): Energy ~8400 kJ/day (women) / ~10,500 kJ/day (men); Calcium 700 mg/day; Protein 45–55 g/day; Fibre 30 g/day; Total fat 70 g/day; Saturated fat <20 g/day; Cholesterol <300 mg/day; Iodine 140 µg/day; Vitamin B12 1.5 µg/day; Vitamin D 10 µg/day. Values are population-level references; individual requirements vary by sex, age, and health status.*  *Abbreviations: Q, quartile; UPF, ultra-processed food; hPDI, healthful plant-based diet index; SD, standard deviation.* | | | | | | |

| **Table S7**. **Key nutrient intakes across quartiles (Q) of high-ultra-processed uPDI (n=124,791)** | | | | | | |
| --- | --- | --- | --- | --- | --- | --- |
|  | **High-UPF uPDI** | | | | **Whole Sample** | |
|  | **Mean (SD)** | | | | **Mean (SD)** | |
| **Key nutrient intakes** | **Q1**  **(n=36,207)** | **Q2**  **(n=33,596)** | **Q3**  **(n=24,487)** | **Q4**  **(n=30,501)** | | **(n=124,791)** |
| Modified Nutrient Quality Index | 13·0 (2·0) | 12·7 (2·0) | 12·3 (2·1) | 11·9 (2·1) | | 12·5 (2·1) |
| Energy, kJ/day | 8444·9 (1919·6) | 8376·7 (1935·9) | 8632·9 (1984·0) | 8779·4 (1984·0) | | 8545·2 (1959·1) |
| Calcium, mg/day | 1044·9 (294·8) | 967·9 (282·2) | 950·5 (280·8) | 915·4 (271·8) | | 974·0 (287·4) |
| Protein, g/day | 81·6 (19·7) | 79·4 (19·3) | 79·8 (19·3) | 79·1 (19·7) | | 80·0 (19·6) |
| Fibre, g/day | 19·9 (6·0) | 18·0 (5·5) | 17·0 (5·3) | 15·8 (5·0) | | 17·8 (5·7) |
| Total fat, g/day | 70·8 (23·1) | 70·7 (22·8) | 73·3 (23·3) | 75·8 (23·0) | | 72·5 (23·1) |
| Saturated fat, g/day | 25·7 (9·8) | 26·2 (9·8) | 27·4 (10·1) | 28·4 (10·0) | | 26·8 (10·0) |
| Cholesterol, mg/day | 175·3 (99·2) | 178·9 (95·3) | 182·8 (94·6) | 181·5 (92·6) | | 179·3 (95·7) |
| Iodine, µg/day | 221·7 (84·5) | 210·1 (83·7) | 206·1 (81·6) | 194·1 (79·9) | | 208·8 (83·2) |
| Vitamin B12, µg/day | 6·4 (2·9) | 6·2 (2·7) | 6·1 (2·6) | 5·8 (2·5) | | 6·1 (2·7) |
| Vitamin D, µg/day | 3·7 (2·5) | 3·7 (2·4) | 3·6 (2·3) | 3·5 (2·2) | | 3·6 (2·4) |
| *Quartiles (Q) are based on index scores, where Q1 represents the lowest adherence and Q4 represents the highest adherence to each dietary index.*  *Recommended nutrient intakes for adults are provided for reference (UK Reference Nutrient Intakes/Dietary Reference Values): Energy ~8400 kJ/day (women) / ~10,500 kJ/day (men); Calcium 700 mg/day; Protein 45–55 g/day; Fibre 30 g/day; Total fat 70 g/day; Saturated fat <20 g/day; Cholesterol <300 mg/day; Iodine 140 µg/day; Vitamin B12 1.5 µg/day; Vitamin D 10 µg/day. Values are population-level references; individual requirements vary by sex, age, and health status.*  *Abbreviations: Q, quartile; UPF, ultra-processed food; uPDI, unhealthful plant-based diet index; SD, standard deviation.* | | | | | | |

| **Table S8**. **Key nutrient intakes across quartiles (Q) of low-ultra-processed uPDI (n=124,791)** | | | | | |
| --- | --- | --- | --- | --- | --- |
|  | **Low-UPF uPDI** | | | | **Whole Sample** |
|  | **Mean (SD)** | | | | **Mean (SD)** |
| **Key nutrient intakes** | **Q1**  **(n=35,354)** | **Q2**  **(n=31,413)** | **Q3**  **(n=29,987)** | **Q4**  **(n=28,037)** | **(n=124,791)** |
| Modified Nutrient Quality Index | 12·7 (2·1) | 12·6 (2·1) | 12·4 (2·1) | 12·3 (2·0) | 12·5 (2·1) |
| Energy, kJ/day | 9169·2 (2001·8) | 8532·4 (1879·2) | 8408·3 (1877·8) | 7919·1 (1843·6) | 8545·2 (1959·1) |
| Calcium, mg/day | 1080·3 (292·5) | 984·1 (271·9) | 940·9 (269·8) | 864·1 (267·5) | 974·0 (287·4) |
| Protein, g/day | 90·0 (19·7) | 80·7 (17·6) | 77·2 (17·5) | 69·7 (17·3) | 80·0 (19·6) |
| Fibre, g/day | 20·5 (5·9) | 18·1 (5·3) | 16·8 (5·2) | 15·2 (4·9) | 17·8 (5·7) |
| Total fat, g/day | 82·3 (24·3) | 73·0 (21·8) | 69·6 (21·2) | 62·7 (20·0) | 72·5 (23·1) |
| Saturated fat, g/day | 29·9 (10·6) | 27·1 (9·7) | 26·0 (9·4) | 23·5 (8·7) | 26·8 (10·0) |
| Cholesterol, mg/day | 226·6 (109·7) | 183·6 (88·8) | 164·0 (80·7) | 131·1 (66·2) | 179·3 (95·7) |
| Iodine, µg/day | 235·5 (88·8) | 211·6 (80·0) | 201·5 (78·3) | 179·8 (73·2) | 208·8 (83·2) |
| Vitamin B12, µg/day | 7·2 (2·9) | 6·3 (2·6) | 5·8 (2·5) | 5·0 (2·3) | 6·1 (2·7) |
| Vitamin D, µg/day | 4·5 (2·7) | 3·7 (2·3) | 3·4 (2·1) | 2·8 (1·9) | 3·6 (2·4) |
| *Quartiles (Q) are based on index scores, where Q1 represents the lowest adherence and Q4 represents the highest adherence to each dietary index.*  *Recommended nutrient intakes for adults are provided for reference (UK Reference Nutrient Intakes/Dietary Reference Values): Energy ~8400 kJ/day (women) / ~10,500 kJ/day (men); Calcium 700 mg/day; Protein 45–55 g/day; Fibre 30 g/day; Total fat 70 g/day; Saturated fat <20 g/day; Cholesterol <300 mg/day; Iodine 140 µg/day; Vitamin B12 1.5 µg/day; Vitamin D 10 µg/day. Values are population-level references; individual requirements vary by sex, age, and health status.*  *Abbreviations: Q, quartile; UPF, ultra-processed food; uPDI, unhealthful plant-based diet index; SD, standard deviation.* | | | | | |

| **Table S9**. **Key nutrient intakes across quartiles (Q) of the ratio of low-UPF: high-UPF hPDI (n=124,791)** | | | | | |
| --- | --- | --- | --- | --- | --- |
|  | **Mean (SD)** | | | |  |
| **Key nutrient intakes** | **Q1**  **(n=31,221)** | **Q2**  **(n=31,320)** | **Q3**  **(n=31,553)** | **Q4**  **(n=30,697)** | **Whole Sample**  **(n=124,791)** |
| Modified Nutrient Quality Index | 12·6 (2·1) | 12·7 (2·1) | 12·6 (2·1) | 12·1 (2·1) | 12·5 (2·1) |
| Energy, kJ/day | 8431·9 (1904·8) | 8311·7 (1925·2) | 8449·1 (1937·3) | 8997·5 (1997·5) | 8545·2 (1959·1) |
| Calcium, mg/day | 932·9 (279·0) | 947·9 (281·4) | 977·4 (284·5) | 1039·0 (293·4) | 974·0 (287·4) |
| Protein, g/day | 82·0 (20·0) | 78·9 (19·5) | 78·8 (19·3) | 80·6 (19·3) | 80·0 (19·6) |
| Fibre, g/day | 17·0 (5·5) | 17·6 (5·7) | 18·1 (5·8) | 18·5 (5·7) | 17·8 (5·7) |
| Total fat, g/day | 72·4 (23·1) | 70·2 (22·8) | 71·1 (22·8) | 76·4 (23·3) | 72·5 (23·1) |
| Saturated fat, g/day | 27·3 (10·2) | 26·0 (9·8) | 26·1 (9·7) | 27·9 (9·9) | 26·8 (10·0) |
| Cholesterol, mg/day | 210·4 (106·5) | 180·1 (95·6) | 167·0 (88·3) | 159·4 (82·6) | 179·3 (95·7) |
| Iodine, µg/day | 204·2 (82·8) | 203·3 (82·2) | 206·5 (81·4) | 221·4 (85·2) | 208·8 (83·2) |
| Vitamin B12, µg/day | 6·5 (2·8) | 6·1 (2·7) | 6·0 (2·7) | 6·0 (2·6) | 6·1 (2·7) |
| Vitamin D, µg/day | 4·2 (2·7) | 3·7 (2·4) | 3·5 (2·3) | 3·2 (2·0) | 3·6 (2·4) |
| *Quartiles (Q) are based on index scores, where Q1 represents the lowest adherence and Q4 represents the highest adherence to each dietary index.*  *Recommended nutrient intakes for adults are provided for reference (UK Reference Nutrient Intakes/Dietary Reference Values): Energy ~8400 kJ/day (women) / ~10,500 kJ/day (men); Calcium 700 mg/day; Protein 45–55 g/day; Fibre 30 g/day; Total fat 70 g/day; Saturated fat <20 g/day; Cholesterol <300 mg/day; Iodine 140 µg/day; Vitamin B12 1.5 µg/day; Vitamin D 10 µg/day. Values are population-level references; individual requirements vary by sex, age, and health status.*  *Abbreviations: Q, quartile; UPF, ultra-processed food; hPDI, healthful plant-based diet index; SD, standard deviation.* | | | | | |

| **Table S10**. **Key nutrient intakes across quartiles (Q) of the ratio of low-UPF: high-UPF uPDI (n=124,791)** | | | | | |
| --- | --- | --- | --- | --- | --- |
|  | **Mean (SD)** | | | |  |
| **Key nutrient intakes** | **Q1**  **(n=31,300)** | **Q2**  **(n=32,008)** | **Q3**  **(n=30,801)** | **Q4**  **(n=30,682)** | **Whole sample**  **(n=124,791)** |
| Modified Nutrient Quality Index | 12·3 (2·2) | 12·4 (2·1) | 12·6 (2·1) | 12·7 (2·0) | 12·5 (2·1) |
| Energy, kJ/day | 9234·8 (1983·1) | 8576·7 (1896·1) | 8272·5 (1877·0) | 8082·8 (1880·5) | 8545·2 (1959·1) |
| Calcium, mg/day | 1030·4 (288·8) | 961·9 (279·5) | 951·8 (282·5) | 951·5 (291·5) | 974·0 (287·4) |
| Protein, g/day | 88·4 (19·9) | 80·2 (18·5) | 77·2 (18·4) | 74·2 (18·6) | 80·0 (19·6) |
| Fibre, g/day | 18·8 (5·9) | 17·4 (5·5) | 17·5 (5·6) | 17·5 (5·6) | 17·8 (5·7) |
| Total fat, g/day | 83·2 (23·6) | 73·4 (21·9) | 68·7 (21·4) | 64·5 (21·2) | 72·5 (23·1) |
| Saturated fat, g/day | 30·6 (10·4) | 27·3 (9·6) | 25·5 (9·4) | 23·9 (9·1) | 26·8 (10·0) |
| Cholesterol, mg/day | 223·7 (107·0) | 184·3 (90·6) | 166·2 (85·3) | 141·9 (78·0) | 179·3 (95·7) |
| Iodine, µg/day | 224·0 (88·0) | 207·5 (81·6) | 203·7 (81·1) | 199·7 (79·8) | 208·8 (83·2) |
| Vitamin B12, µg/day | 6·9 (2·8) | 6·2 (2·6) | 5·9 (2·6) | 5·5 (2·6) | 6·1 (2·7) |
| Vitamin D, µg/day | 4·3 (2·6) | 3·7 (2·3) | 3·5 (2·3) | 3·0 (2·1) | 3·6 (2·4) |
| *Quartiles (Q) are based on index scores, where Q1 represents the lowest adherence and Q4 represents the highest adherence to each dietary index.*  *Recommended nutrient intakes for adults are provided for reference (UK Reference Nutrient Intakes/Dietary Reference Values): Energy ~8400 kJ/day (women) / ~10,500 kJ/day (men); Calcium 700 mg/day; Protein 45–55 g/day; Fibre 30 g/day; Total fat 70 g/day; Saturated fat <20 g/day; Cholesterol <300 mg/day; Iodine 140 µg/day; Vitamin B12 1.5 µg/day; Vitamin D 10 µg/day. Values are population-level references; individual requirements vary by sex, age, and health status.*  *Abbreviations: Q, quartile; UPF, ultra-processed food; uPDI, unhealthful plant-based diet index; SD, standard deviation.* | | | | | |

| **Table S11.** **Hazard ratios (95% confidence intervals) of mortality and major chronic diseases across sex-specific quartiles (Q) of high-ultra-processed and low-ultra-processed hPDI and uPDI** | | | | | | | | | | |
| --- | --- | --- | --- | --- | --- | --- | --- | --- | --- | --- |
|  | **High-UPF hPDI** | | | | P-trend | **Low-UPF hPDI** | | | | P-trend |
|  | Q1 | Q2 | Q3 | Q4 |  | Q1 | Q2 | Q3 | Q4 |  |
| **All-cause mortality** |  |  |  |  |  |  |  |  |  |  |
| Cases/total | 1,799/37,692 | 1,354/30,041 | 1,504/32,670 | 1,123/24,388 |  | 1,474/32,136 | 1,656/35,856 | 1,422/28,767 | 1,228/28,032 |  |
| HR (95% CI) |  |  |  |  |  |  |  |  |  |  |
| Model 1 | 1·00^a^ | 0·86 (0·81-0·93) | 0·82 (0·76-0·88) | 0·83 (0·77-0·89) | <0·001 | 1·00^a^ | 0·95 (0·88-1·02) | 0·93 (0·87-1·00) | 0·86 (0·80-0·93) | <0·001 |
| Model 2 | 1·00^a^ | 0·91 (0·85-0·98) | 0·89 (0·83-0·96) | 0·92 (0·85-1·00) | 0·002 | 1·00^a^ | 0·97 (0·91-1·05) | 0·97 (0·90-1·04) | 0·91 (0·84-0·98) | 0·002 |
| **T2DM** |  |  |  |  |  |  |  |  |  |  |
| Cases/total | 1,253/35,926 | 795/28,894 | 823/31,358 | 549/23,452 |  | 1,042/30,862 | 938/34,466 | 814/27,506 | 626/26,796 |  |
| HR (95% CI) |  |  |  |  |  |  |  |  |  |  |
| Model 1 | 1·00^a^ | 0·76 (0·70-0·83) | 0·69 (0·63-0·75) | 0·62 (0·56-0·69) | <0·001 | 1·00^a^ | 0·76 (0·69-0·83) | 0·76 (0·69-0·83) | 0·62 (0·56-0·69) | <0·001 |
| Model 2 | 1·00^a^ | 0·91 (0·83-1·00) | 0·91 (0·82-1·00) | 0·89 (0·79-0·99) | 0·001 | 1·00^a^ | 0·80 (0·73-0·88) | 0·83 (0·76-0·91) | 0·72 (0·65-0·79) | <0·001 |
| **CVD** |  |  |  |  |  |  |  |  |  |  |
| Cases/total | 1,839/35,892 | 1,464/28,639 | 1,633/31,070 | 1,142/23,273 |  | 1,648/32,999 | 1,510/30,894 | 1,533/27,677 | 1,387/27,304 |  |
| HR (95% CI) |  |  |  |  |  |  |  |  |  |  |
| Model 1 | 1·00^a^ | 0·93 (0·87-0·99) | 0·88 (0·82-0·94) | 0·83 (0·77-0·90) | <0·001 | 1·00^a^ | 1·00 (0·93-1·08) | 0·99 (0·93-1·07) | 0·96 (0·89-1·03) | 0·14 |
| Model 2 | 1·00^a^ | 0·96 (0·90-1·03) | 0·93 (0·87-1·00) | 0·89 (0·82-0·96) | 0·001 | 1·00^a^ | 1·01 (0·95-1·09) | 1·00 (0·94-1·08) | 0·98 (0·91-1·05) | 0·39 |
| **Cancer** |  |  |  |  |  |  |  |  |  |  |
| Cases/total | 2,793/34,046 | 2,225/26,939 | 2,585/29,350 | 1,834/21,870 |  | 2,547/31,093 | 2,439/28,932 | 2,355/26,300 | 2,096/25,880 |  |
| HR (95% CI) |  |  |  |  |  |  |  |  |  |  |
| Model 1 | 1·00^a^ | 0·93 (0·88-0·99) | 0·94 (0·89-0·99) | 0·89 (0·84-0·94) | <0·001 | 1·00^a^ | 1·03 (0·97-1·09) | 1·02 (0·96-1·07) | 0·93 (0·88-0·99) | 0·005 |
| Model 2 | 1·00^a^ | 0·96 (0·90-1·01) | 0·98 (0·92-1·03) | 0·95 (0·89-1·01) | 0·15 | 1·00^a^ | 1·05 (0·99-1·11) | 1·04 (0·98-1·10) | 0·97 (0·91-1·03) | 0·19 |
|  | **High-UPF uPDI** | | | | P-trend | **Low-UPF uPDI** | | | | P-trend |
|  | Q1 | Q2 | Q3 | Q4 |  | Q1 | Q2 | Q3 | Q4 |  |
| **All-cause mortality** |  |  |  |  |  |  |  |  |  |  |
| Cases/total | 1,650/36,207 | 1,508/33,596 | 1,141/24,487 | 1,481/30,501 |  | 1,703/35,354 | 1,419/31,413 | 1,380/29,987 | 1,278/28,037 |  |
| HR (95% CI) |  |  |  |  |  |  |  |  |  |  |
| Model 1 | 1·00^a^ | 1·01 (0·95-1·09) | 1·03 (0·96-1·11) | 1·22 (1·13-1·31) | <0·001 | 1·00^a^ | 1·03 (0·96-1·10) | 1·01 (0·94-1·09) | 1·14 (1·06-1·23) | 0·003 |
| Model 2 | 1·00^a^ | 1·00 (0·93-1·08) | 0·99 (0·92-1·07) | 1·09 (1·01-1·17) | 0·02 | 1·00^a^ | 1·03 (0·96-1·11) | 1·01 (0·94-1·09) | 1·13 (1·05-1·22) | 0·008 |
| **T2DM** |  |  |  |  |  |  |  |  |  |  |
| Cases/total | 880/34,831 | 834/32,332 | 699/23,449 | 1,007/29,018 |  | 880/33,593 | 976/34,013 | 729/25,024 | 835/27,000 |  |
| HR (95% CI) |  |  |  |  |  |  |  |  |  |  |
| Model 1 | 1·00^a^ | 1·03 (0·93-1·13) | 1·12 (1·01-1·24) | 1·40 (1·28-1·54) | <0·001 | 1·00^a^ | 1·12 (1·02-1·23) | 1·17 (1·06-1·29) | 1·29 (1·18-1·42) | <0·001 |
| Model 2 | 1·00^a^ | 1·01 (0·92-1·11) | 1·04 (0·94-1·15) | 1·12 (1·02-1·23) | 0·01 | 1·00^a^ | 1·14 (1·04-1·26) | 1·19 (1·07-1·32) | 1·31 (1·18-1·45) | <0·001 |
| **CVD** |  |  |  |  |  |  |  |  |  |  |
| Cases/total | 1,733/34,592 | 1,572/32,109 | 1,265/23,198 | 1,508/28,975 |  | 1,912/34,488 | 1,457/30,373 | 1,508/28,459 | 1,201/25,554 |  |
| HR (95% CI) |  |  |  |  |  |  |  |  |  |  |
| Model 1 | 1·00^a^ | 1·00 (0·94-1·08) | 1·07 (0·99-1·15) | 1·16 (1·08-1·24) | <0·001 | 1·00^a^ | 0·95 (0·89-1·02) | 0·98 (0·92-1·05) | 1·00 (0·93-1·08) | 0·68 |
| Model 2 | 1·00^a^ | 1·00 (0·94-1·07) | 1·05 (0·98-1·13) | 1·07 (1·00-1·15) | 0·02 | 1·00^a^ | 0·95 (0·89-1·02) | 0·97 (0·90-1·04) | 0·97 (0·90-1·05) | 0·59 |
| **Cancer** |  |  |  |  |  |  |  |  |  |  |
| Cases/total | 2,779/32,438 | 2,643/30,095 | 1,826/22,068 | 2,189/27,604 |  | 2,814/32,400 | 2,390/28,458 | 2,286/27,036 | 1,947/24,311 |  |
| HR (95% CI) |  |  |  |  |  |  |  |  |  |  |
| Model 1 | 1·00^a^ | 1·06 (1·00-1·12) | 0·99 (0·94-1·05) | 1·05 (0·99-1·11) | 0·15 | 1·00^a^ | 1·03 (0·98-1·09) | 1·02 (0·97-1·08) | 1·07 (1·01-1·13) | 0·07 |
| Model 2 | 1·00^a^ | 1·05 (0·99-1·10) | 0·97 (0·91-1·03) | 1·00 (0·94-1·06) | 0·65 | 1·00^a^ | 1·04 (0·99-1·10) | 1·03 (0·97-1·09) | 1·09 (1·02-1·16) | 0·03 |
| Model 1 adjusted for sex, education, Townsend deprivation index, and ethnicity; stratified by age (5-year categories) and region.  Model 2: Model 1 plus BMI, physical activity, smoking status, alcohol intake, energy intake, multimorbidity index, polypharmacy, blood pressure medications, blood thinning medications, cholesterol lowering medications, and number of completed dietary assessments. All-cause mortality models also adjusted for CVD and cancer at baseline; T2DM models also adjusted for CVD and cancer at baseline; CVD models also adjusted for T2DM and cancer at baseline; cancer models also adjusted for CVD and T2DM at baseline, menopausal status and menopause hormone treatment. For high-UPF hPDI and high-UPF uPDI analyses, models also adjusted intake of fruit, nuts, animal-fat, fruit juice and eggs. For low-UPF hPDI and low-UPF uPDI, models also adjusted for intake of sugar-sweetened beverages, sweets and desserts and miscellaneous animal-based foods.  P-trend is for linear trend.  ^a^Reference categories.  *Abbreviations: Q, quartile; UPF, ultra-processed food; PDI, plant-based diet index; hPDI, healthful plant-based diet index; uPDI, unhealthful plant-based diet index; BMI, Body Mass Index; HR, hazard ratio; CI, confidence interval; T2DM, type 2 diabetes mellitus; CVD, cardiovascular disease.* | | | | | | | | | | |

| **Table S12.** **Hazard ratios (95% confidence intervals) of all-cause mortality (n=124,791), T2DM (n=119,630), CVD (n=118,874), and cancer (n=112,205) across high-ultra-processed and low-ultra-processed PDI food groups** | | | | |
| --- | --- | --- | --- | --- |
|  | **High-UPF Food items**  **(servings/day)** | | **Low-UPF Food Items**  **(servings/day)** | |
|  | **HR (95% CI)** | **P-trend** | **HR (95% CI)** | **P-trend** |
| **All-cause mortality** |  |  |  |  |
| Wholegrains | 0·97 (0·95-0·99) | 0·001 | 0·95 (0·88-1·02) | 0·18 |
| Fruit |  |  | 0·98 (0·96-1·00) | 0·02 |
| Vegetables | 1·02 (0·81-1·28) | 0·89 | 0·98 (0·96-0·99) | 0·006 |
| Nuts |  |  | 0·91 (0·84-0·99) | 0·03 |
| Legumes & vegetarian protein alternatives | 1·02 (0·92-1·13) | 0·71 | 0·96 (0·89-1·03) | 0·24 |
| Tea and coffee | 0·97 (0·81-1·17) | 0·76 | 0·97 (0·95-0·99) | <0·001 |
| Refined grains | 1·04 (1·01-1·07) | 0·002 | 0·88 (0·80-0·97) | 0·01 |
| Potatoes | 1·08 (1·01-1·15) | 0·02 | 1·00 (0·94-1·07) | 0·94 |
| Sugar-sweetened beverages | 1·07 (1·04-1·10) | <0·001 |  |  |
| Fruit juice |  |  | 1·00 (0·95-1·05) | 0·94 |
| Sweets and desserts | 1·01 (0·99-1·04) | 0·26 |  |  |
| Animal fat |  |  | 1·01 (1·00-1·02) | 0·007 |
| Dairy | 1·03 (0·98-1·07) | 0·29 | 1·02 (0·97-1·08) | 0·42 |
| Eggs |  |  | 0·98 (0·92-1·04) | 0·44 |
| Fish or seafood | 1·13 (0·98-1·30) | 0·11 | 0·92 (0·86-0·99) | 0·02 |
| Meat | 1·02 (0·97-1·06) | 0·52 | 1·02 (0·97-1·07) | 0·46 |
| Miscellaneous animal-based foods | 0·98 (0·89-1·08) | 0·69 |  |  |
| **T2DM** |  |  |  |  |
| Wholegrains | 0·99 (0·97-1·01) | 0·33 | 0·86 (0·77-0·95) | 0·004 |
| Fruit |  |  | 0·96 (0·94-0·98) | <0·001 |
| Vegetables | 1·09 (0·82-1·45) | 0·55 | 0·96 (0·95-0·98) | <0·001 |
| Nuts |  |  | 0·84 (0·75-0·94) | 0·003 |
| Legumes & vegetarian protein alternatives | 1·06 (0·93-1·20) | 0·40 | 0·93 (0·84-1·02) | 0·14 |
| Tea and coffee | 1·00 (0·80-1·23) | 0·96 | 0·91 (0·89-0·93) | <0·001 |
| Refined grains | 1·07 (1·04-1·10) | <0·001 | 0·97 (0·86-1·10) | 0·61 |
| Potatoes | 1·15 (1·07-1·25) | <0·001 | 1·05 (0·97-1·15) | 0·25 |
| Sugar-sweetened beverages | 1·13 (1·09-1·17) | <0·001 |  |  |
| Fruit juice |  |  | 0·99 (0·93-1·06) | 0·81 |
| Sweets and desserts | 1·01 (0·98-1·04) | 0·70 |  |  |
| Animal fat |  |  | 1·01 (1·00-1·02) | 0·15 |
| Dairy | 0·93 (0·88-0·99) | 0·02 | 1·04 (0·96-1·11) | 0·34 |
| Eggs |  |  | 1·01 (0·93-1·08) | 0·89 |
| Fish or seafood | 1·05 (0·88-1·26) | 0·58 | 0·86 (0·78-0·95) | 0·002 |
| Meat | 1·11 (1·05-1·16) | <0·001 | 1·10 (1·04-1·16) | 0·001 |
| Miscellaneous animal-based foods | 1·08 (0·97-1·21) | 0·18 |  |  |
| **CVD** |  |  |  |  |
| Wholegrains | 0·98 (0·97-1·00) | 0·08 | 0·94 (0·87-1·01) | 0·11 |
| Fruit |  |  | 0·99 (0·97-1·00) | 0·12 |
| Vegetables | 1·14 (0·92-1·42) | 0·23 | 0·99 (0·98-1·01) | 0·20 |
| Nuts |  |  | 0·92 (0·85-1·00) | 0·05 |
| Legumes & vegetarian protein alternatives | 0·96 (0·87-1·07) | 0·48 | 0·96 (0·90-1·03) | 0·29 |
| Tea and coffee | 1·01 (0·85-1·21) | 0·90 | 0·99 (0·98-1·01) | 0·21 |
| Refined grains | 1·03 (1·00-1·05) | 0·03 | 0·87 (0·79-0·95) | 0·004 |
| Potatoes | 1·07 (1·01-1·14) | 0·03 | 0·99 (0·93-1·06) | 0·76 |
| Sugar-sweetened beverages | 1·09 (1·06-1·12) | <0·001 |  |  |
| Fruit juice |  |  | 0·98 (0·93-1·03) | 0·40 |
| Sweets and desserts | 1·02 (1·00-1·04) | 0·10 |  |  |
| Animal fat |  |  | 1·01 (1·00-1·02) | 0·02 |
| Dairy | 1·00 (0·96-1·05) | 0·90 | 0·92 (0·87-0·98) | 0·005 |
| Eggs |  |  | 1·00 (0·95-1·06) | 0·95 |
| Fish or seafood | 1·12 (0·97-1·29) | 0·12 | 0·98 (0·91-1·05) | 0·54 |
| Meat | 1·02 (0·98-1·06) | 0·41 | 1·06 (1·01-1·11) | 0·01 |
| Miscellaneous animal-based foods | 0·97 (0·88-1·06) | 0·51 |  |  |
| **Cancer** |  |  |  |  |
| Wholegrains | 1·00 (0·98-1·01) | 0·50 | 0·98 (0·93-1·04) | 0·59 |
| Fruit |  |  | 0·99 (0·97-1·00) | 0·03 |
| Vegetables | 0·94 (0·79-1·13) | 0·53 | 0·99 (0·98-1·00) | 0·08 |
| Nuts |  |  | 0·97 (0·91-1·03) | 0·27 |
| Legumes & vegetarian protein alternatives | 0·95 (0·87-1·03) | 0·22 | 0·91 (0·86-0·96) | 0·001 |
| Tea and coffee | 1·15 (1·00-1·32) | 0·05 | 0·99 (0·98-1·01) | 0·23 |
| Refined grains | 1·01 (0·99-1·03) | 0·44 | 0·98 (0·91-1·06) | 0·60 |
| Potatoes | 1·04 (0·99-1·10) | 0·11 | 0·98 (0·92-1·03) | 0·38 |
| Sugar-sweetened beverages | 1·00 (0·97-1·02) | 0·70 |  |  |
| Fruit juice |  |  | 0·98 (0·94-1·02) | 0·27 |
| Sweets and desserts | 1·01 (0·99-1·03) | 0·24 |  |  |
| Animal fat |  |  | 1·00 (0·99-1·01) | 0·94 |
| Dairy | 1·02 (0·99-1·06) | 0·25 | 0·99 (0·95-1·04) | 0·73 |
| Eggs |  |  | 0·98 (0·93-1·03) | 0·35 |
| Fish or seafood | 1·09 (0·96-1·22) | 0·18 | 0·96 (0·91-1·02) | 0·17 |
| Meat | 1·01 (0·97-1·04) | 0·69 | 0·99 (0·96-1·03) | 0·63 |
| Miscellaneous animal-based foods | 1·06 (0·98-1·13) | 0·14 |  |  |
| Hazard Ratios with 95% Confidence Intervals (CI) are presented per one serving per day increase in each food group. HRs are adjusted for sex, education, ethnicity, BMI, physical activity, smoking status, alcohol intake, energy intake, multimorbidity index, polypharmacy, blood pressure medications, blood thinning medications, cholesterol lowering medications, Townsend deprivation index, and number of completed dietary assessments; stratified by age (5-year categories) and region. All-cause mortality models also adjusted for CVD and cancer at baseline; T2DM models also adjusted for CVD and cancer at baseline; CVD models also adjusted for T2DM and cancer at baseline; cancer models also adjusted for CVD and T2DM at baseline, menopausal status and menopause hormone treatment.  P-trend is for linear trend.  *Abbreviations: UPF, ultra-processed food; PDI, plant-based diet index; BMI, Body Mass Index; HR, hazard ratio; CI, confidence interval; T2DM, type 2 diabetes mellitus; CVD, cardiovascular disease.* | | | | |

| **Table S13.** **Hazard ratios (95% confidence intervals) of all-cause mortality (n=124,791), T2DM (n=119,630), CVD (n=118,874), and cancer (n=112,205) across high-ultra-processed and low-ultra-processed hPDI (10-point increments), systematically excluding each food group** | | | | |
| --- | --- | --- | --- | --- |
|  | **High-UPF hPDI**  **(10-point increments)** | | **Low-UPF hPDI**  **(10-point increments)** | |
|  | **HR (95% CI)** | **P-trend** | **HR (95% CI)** | **P-trend** |
| **All-cause mortality** |  |  |  |  |
| Excluding wholegrains | 0·92 (0·86-0·99) | 0·02 | 0·92 (0·88-0·98) | 0·004 |
| Excluding fruit |  |  | 0·93 (0·88-0·99) | 0·01 |
| Excluding vegetables | 0·91 (0·85-0·97) | 0·002 | 0·94 (0·89-0·99) | 0·02 |
| Excluding nuts |  |  | 0·94 (0·89-0·99) | 0·01 |
| Excluding legumes & vegetarian protein alternatives | 0·89 (0·84-0·95) | 0·001 | 0·92 (0·88-0·98) | 0·004 |
| Excluding tea and coffee | 0·91 (0·85-0·97) | 0·002 | 0·93 (0·88-0·98) | 0·007 |
| Excluding refined grains | 0·91 (0·85-0·97) | 0·005 | 0·91 (0·86-0·95) | <0·001 |
| Excluding potatoes | 0·91 (0·85-0·97) | 0·005 | 0·93 (0·88-0·97) | 0·003 |
| Excluding sugar-sweetened beverages | 0·93 (0·87-1·00) | 0·04 |  |  |
| Excluding fruit juice |  |  | 0·92 (0·87-0·97) | 0·001 |
| Excluding sweets and desserts | 0·89 (0·84-0·95) | 0·001 |  |  |
| Excluding animal fat |  |  | 0·93 (0·88-0·99) | 0·01 |
| Excluding dairy | 0·91 (0·86-0·97) | 0·005 | 0·93 (0·88-0·98) | 0·004 |
| Excluding eggs |  |  | 0·92 (0·87-0·97) | 0·001 |
| Excluding fish or seafood | 0·91 (0·85-0·97) | 0·004 | 0·91 (0·87-0·96) | <0·001 |
| Excluding meat | 0·89 (0·83-0·95) | 0·001 | 0·92 (0·87-0·97) | 0·001 |
| Excluding miscellaneous animal-based foods | 0·90 (0·85-0·96) | 0·001 |  |  |
| **T2DM** |  |  |  |  |
| Excluding wholegrains | 0·86 (0·78-0·93) | 0·001 | 0·81 (0·76-0·87) | <0·001 |
| Excluding fruit |  |  | 0·81 (0·76-0·87) | <0·001 |
| Excluding vegetables | 0·86 (0·79-0·93) | <0·001 | 0·83 (0·78-0·89) | <0·001 |
| Excluding nuts |  |  | 0·82 (0·76-0·87) | <0·001 |
| Excluding legumes & vegetarian protein alternatives | 0·84 (0·77-0·91) | <0·001 | 0·81 (0·75-0·86) | <0·001 |
| Excluding tea and coffee | 0·87 (0·80-0·94) | <0·001 | 0·85 (0·79-0·91) | <0·001 |
| Excluding refined grains | 0·88 (0·81-0·96) | 0·005 | 0·80 (0·75-0·86) | <0·001 |
| Excluding potatoes | 0·88 (0·81-0·96) | 0·003 | 0·83 (0·78-0·89) | <0·001 |
| Excluding sugar-sweetened beverages | 0·94 (0·86-1·02) | 0·13 |  |  |
| Excluding fruit juice |  |  | 0·80 (0·75-0·86) | <0·001 |
| Excluding sweets and desserts | 0·86 (0·79-0·93) | <0·001 |  |  |
| Excluding animal fat |  |  | 0·80 (0·75-0·86) | <0·001 |
| Excluding dairy | 0·83 (0·77-0·90) | <0·001 | 0·82 (0·77-0·88) | <0·001 |
| Excluding eggs |  |  | 0·80 (0·75-0·86) | <0·001 |
| Excluding fish or seafood | 0·87 (0·80-0·94) | 0·001 | 0·79 (0·74-0·84) | <0·001 |
| Excluding meat | 0·90 (0·82-0·98) | 0·01 | 0·83 (0·77-0·88) | <0·001 |
| Excluding miscellaneous animal-based foods | 0·88 (0·81-0·95) | 0·001 |  |  |
| **CVD** |  |  |  |  |
| Excluding wholegrains | 0·90 (0·84-0·97) | 0·003 | 0·99 (0·94-1·04) | 0·61 |
| Excluding fruit |  |  | 0·98 (0·93-1·04) | 0·50 |
| Excluding vegetables | 0·89 (0·84-0·95) | <0·001 | 0·98 (0·93-1·03) | 0·49 |
| Excluding nuts |  |  | 0·98 (0·93-1·04) | 0·54 |
| Excluding legumes & vegetarian protein alternatives | 0·90 (0·84-0·95) | 0·001 | 0·98 (0·93-1·03) | 0·48 |
| Excluding tea and coffee | 0·90 (0·85-0·96) | 0·001 | 0·98 (0·93-1·03) | 0·38 |
| Excluding refined grains | 0·89 (0·83-0·95) | 0·001 | 0·96 (0·91-1·01) | 0·12 |
| Excluding potatoes | 0·91 (0·86-0·97) | 0·006 | 0·98 (0·93-1·03) | 0·32 |
| Excluding sugar-sweetened beverages | 0·93 (0·87-0·99) | 0·03 |  |  |
| Excluding fruit juice |  |  | 0·97 (0·92-1·02) | 0·23 |
| Excluding sweets and desserts | 0·90 (0·85-0·96) | 0·001 |  |  |
| Excluding animal fat |  |  | 0·99 (0·94-1·05) | 0·78 |
| Excluding dairy | 0·90 (0·84-0·96) | 0·001 | 0·97 (0·92-1·02) | 0·18 |
| Excluding eggs |  |  | 0·98 (0·93-1·03) | 0·44 |
| Excluding fish or seafood | 0·91 (0·85-0·96) | 0·002 | 0·97 (0·93-1·02) | 0·31 |
| Excluding meat | 0·90 (0·85-0·96) | 0·002 | 0·99 (0·94-1·04) | 0·77 |
| Excluding miscellaneous animal-based foods | 0·90 (0·85-0·96) | 0·001 |  |  |
| **Cancer** |  |  |  |  |
| Excluding wholegrains | 0·96 (0·91-1·02) | 0·17 | 0·97 (0·93-1·02) | 0·20 |
| Excluding fruit |  |  | 0·98 (0·94-1·02) | 0·27 |
| Excluding vegetables | 0·97 (0·92-1·02) | 0·17 | 0·98 (0·94-1·02) | 0·39 |
| Excluding nuts |  |  | 0·98 (0·94-1·02) | 0·23 |
| Excluding legumes & vegetarian protein alternatives | 0·97 (0·92-1·02) | 0·24 | 0·99 (0·95-1·03) | 0·53 |
| Excluding tea and coffee | 0·96 (0·92-1·01) | 0·11 | 0·98 (0·94-1·02) | 0·26 |
| Excluding refined grains | 0·96 (0·91-1·01) | 0·09 | 0·97 (0·93-1·01) | 0·18 |
| Excluding potatoes | 0·97 (0·92-1·02) | 0·29 | 0·97 (0·93-1·01) | 0·15 |
| Excluding sugar-sweetened beverages | 0·95 (0·90-1·00) | 0·07 |  |  |
| Excluding fruit juice |  |  | 0·97 (0·93-1·01) | 0·13 |
| Excluding sweets and desserts | 0·96 (0·91-1·01) | 0·13 |  |  |
| Excluding animal fat |  |  | 0·97 (0·93-1·01) | 0·19 |
| Excluding dairy | 0·98 (0·93-1·03) | 0·40 | 0·98 (0·94-1·02) | 0·26 |
| Excluding eggs |  |  | 0·96 (0·92-1·01) | 0·08 |
| Excluding fish or seafood | 0·97 (0·92-1·02) | 0·21 | 0·97 (0·93-1·01) | 0·12 |
| Excluding meat | 0·96 (0·91-1·01) | 0·12 | 0·97 (0·93-1·01) | 0·16 |
| Excluding miscellaneous animal-based foods | 0·97 (0·92-1·02) | 0·19 |  |  |
| Hazard Ratios with 95% Confidence Intervals (CI) are presented per 10-point increase in the dietary index, reflecting a meaningful increase in adherence. HRs are adjusted for sex, education, ethnicity, BMI, physical activity, smoking status, alcohol intake, energy intake, multimorbidity index, polypharmacy, blood pressure medications, blood thinning medications, cholesterol lowering medications, Townsend deprivation index, and number of completed dietary assessments; stratified by age (5-year categories) and region. All-cause mortality models also adjusted for CVD and cancer at baseline; T2DM models also adjusted for CVD and cancer at baseline; CVD models also adjusted for T2DM and cancer at baseline; cancer models also adjusted for CVD and T2DM at baseline, menopausal status and menopause hormone treatment. For high-UPF hPDI analyses, models also adjusted intake of fruit, nuts, animal-fat, fruit juice and eggs. For low-UPF hPDI, models also adjusted for intake of sugar-sweetened beverages, sweets and desserts and miscellaneous animal-based foods.  P-trend is for linear trend.  *Abbreviations: UPF, ultra-processed food; hPDI, healthful plant-based diet index; BMI, Body Mass Index; HR, hazard ratio; CI, confidence interval; T2DM, type 2 diabetes mellitus; CVD, cardiovascular disease.* | | | | |

| **Table S14.** **Hazard ratios (95% confidence intervals) of all-cause mortality (n=124,791), T2DM (n=119,630), CVD (n=118,874), and cancer (n=112,205) across high-ultra-processed and low-ultra-processed uPDI (10-point increments), systematically excluding each food group** | | | | |
| --- | --- | --- | --- | --- |
|  | **High-UPF uPDI**  **(10-point increments)** | | **Low-UPF uPDI**  **(10-point increments)** | |
|  | **HR (95% CI)** | **P-trend** | **HR (95% CI)** | **P-trend** |
| **All-cause mortality** |  |  |  |  |
| Excluding wholegrains | 1·06 (0·99-1·13) | 0·11 | 1·07 (1·01-1·13) | 0·02 |
| Excluding fruit |  |  | 1·06 (1·00-1·12) | 0·05 |
| Excluding vegetables | 1·08 (1·02-1·15) | 0·02 | 1·06 (1·00-1·12) | 0·06 |
| Excluding nuts |  |  | 1·06 (1·00-1·12) | 0·05 |
| Excluding legumes & vegetarian protein alternatives | 1·09 (1·03-1·17) | 0·006 | 1·07 (1·01-1·13) | 0·02 |
| Excluding tea and coffee | 1·08 (1·02-1·15) | 0·01 | 1·07 (1·01-1·13) | 0·03 |
| Excluding refined grains | 1·08 (1·01-1·16) | 0·03 | 1·11 (1·05-1·17) | 0·001 |
| Excluding potatoes | 1·08 (1·01-1·15) | 0·03 | 1·08 (1·02-1·14) | 0·009 |
| Excluding sugar-sweetened beverages | 1·05 (0·98-1·12) | 0·18 |  |  |
| Excluding fruit juice |  |  | 1·09 (1·03-1·16) | 0·003 |
| Excluding sweets and desserts | 1·10 (1·03-1·17) | 0·004 |  |  |
| Excluding animal fat |  |  | 1·10 (1·04-1·17) | <0·001 |
| Excluding dairy | 1·10 (1·03-1·18) | 0·003 | 1·09 (1·03-1·15) | 0·003 |
| Excluding eggs |  |  | 1·09 (1·03-1·15) | 0·005 |
| Excluding fish or seafood | 1·09 (1·02-1·16) | 0·007 | 1·06 (1·01-1·13) | 0·03 |
| Excluding meat | 1·08 (1·02-1·15) | 0·02 | 1·07 (1·02-1·13) | 0·01 |
| Excluding miscellaneous animal-based foods | 1·08 (1·01-1·15) | 0·02 |  |  |
| **T2DM** |  |  |  |  |
| Excluding wholegrains | 1·11 (1·02-1·21) | 0·01 | 1·18 (1·09-1·27) | <0·001 |
| Excluding fruit |  |  | 1·17 (1·09-1·26) | <0·001 |
| Excluding vegetables | 1·12 (1·04-1·22) | 0·005 | 1·15 (1·07-1·24) | <0·001 |
| Excluding nuts |  |  | 1·17 (1·09-1·26) | <0·001 |
| Excluding legumes & vegetarian protein alternatives | 1·14 (1·05-1·24) | 0·002 | 1·19 (1·10-1·28) | <0·001 |
| Excluding tea and coffee | 1·11 (1·03-1·20) | 0·008 | 1·13 (1·05-1·21) | 0·002 |
| Excluding refined grains | 1·09 (1·00-1·19) | 0·06 | 1·21 (1·12-1·30) | <0·001 |
| Excluding potatoes | 1·09 (1·00-1·19) | 0·04 | 1·16 (1·08-1·25) | <0·001 |
| Excluding sugar-sweetened beverages | 1·03 (0·94-1·12) | 0·54 |  |  |
| Excluding fruit juice |  |  | 1·21 (1·13-1·31) | <0·001 |
| Excluding sweets and desserts | 1·13 (1·04-1·23) | 0·004 |  |  |
| Excluding animal fat |  |  | 1·21 (1·13-1·31) | <0·001 |
| Excluding dairy | 1·08 (0·99-1·17) | 0·08 | 1·22 (1·14-1·31) | <0·001 |
| Excluding eggs |  |  | 1·22 (1·13-1·31) | <0·001 |
| Excluding fish or seafood | 1·11 (1·03-1·20) | 0·009 | 1·16 (1·08-1·25) | <0·001 |
| Excluding meat | 1·17 (1·08-1·27) | <0·001 | 1·22 (1·14-1·31) | <0·001 |
| Excluding miscellaneous animal-based foods | 1·12 (1·03-1·21) | 0·005 |  |  |
| **CVD** |  |  |  |  |
| Excluding wholegrains | 1·07 (1·00-1·14) | 0·06 | 0·97 (0·92-1·03) | 0·31 |
| Excluding fruit |  |  | 0·98 (0·93-1·03) | 0·41 |
| Excluding vegetables | 1·09 (1·02-1·15) | 0·009 | 0·98 (0·92-1·03) | 0·42 |
| Excluding nuts |  |  | 0·98 (0·92-1·03) | 0·39 |
| Excluding legumes & vegetarian protein alternatives | 1·08 (1·02-1·15) | 0·01 | 0·98 (0·92-1·03) | 0·43 |
| Excluding tea and coffee | 1·08 (1·01-1·14) | 0·02 | 0·98 (0·93-1·04) | 0·57 |
| Excluding refined grains | 1·08 (1·01-1·16) | 0·02 | 1·01 (0·95-1·06) | 0·86 |
| Excluding potatoes | 1·06 (0·99-1·13) | 0·08 | 0·99 (0·94-1·04) | 0·69 |
| Excluding sugar-sweetened beverages | 1·04 (0·97-1·11) | 0·27 |  |  |
| Excluding fruit juice |  |  | 0·99 (0·94-1·05) | 0·81 |
| Excluding sweets and desserts | 1·08 (1·01-1·15) | 0·02 |  |  |
| Excluding animal fat |  |  | 1·00 (0·95-1·06) | 0·92 |
| Excluding dairy | 1·09 (1·02-1·16) | 0·01 | 0·97 (0·92-1·03) | 0·30 |
| Excluding eggs |  |  | 0·99 (0·93-1·05) | 0·66 |
| Excluding fish or seafood | 1·08 (1·02-1·15) | 0·009 | 0·98 (0·93-1·04) | 0·46 |
| Excluding meat | 1·09 (1·03-1·16) | 0·005 | 1·00 (0·95-1·06) | 0·95 |
| Excluding miscellaneous animal-based foods | 1·07 (1·01-1·14) | 0·02 |  |  |
| **Cancer** |  |  |  |  |
| Excluding wholegrains | 0·98 (0·93-1·04) | 0·48 | 1·05 (1·01-1·10) | 0·03 |
| Excluding fruit |  |  | 1·05 (1·00-1·09) | 0·05 |
| Excluding vegetables | 0·99 (0·94-1·04) | 0·59 | 1·04 (1·00-1·09) | 0·08 |
| Excluding nuts |  |  | 1·05 (1·00-1·10) | 0·04 |
| Excluding legumes & vegetarian protein alternatives | 0·98 (0·93-1·03) | 0·44 | 1·04 (0·99-1·08) | 0·14 |
| Excluding tea and coffee | 0·99 (0·95-1·04) | 0·76 | 1·05 (1·00-1·10) | 0·04 |
| Excluding refined grains | 0·99 (0·94-1·04) | 0·68 | 1·05 (1·01-1·10) | 0·02 |
| Excluding potatoes | 0·98 (0·93-1·03) | 0·34 | 1·05 (1·01-1·10) | 0·02 |
| Excluding sugar-sweetened beverages | 0·99 (0·94-1·05) | 0·82 |  |  |
| Excluding fruit juice |  |  | 1·06 (1·01-1·11) | 0·01 |
| Excluding sweets and desserts | 0·99 (0·94-1·04) | 0·67 |  |  |
| Excluding animal fat |  |  | 1·05 (1·01-1·10) | 0·02 |
| Excluding dairy | 1·01 (0·95-1·06) | 0·86 | 1·06 (1·01-1·10) | 0·01 |
| Excluding eggs |  |  | 1·04 (1·00-1·09) | 0·06 |
| Excluding fish or seafood | 0·99 (0·95-1·04) | 0·81 | 1·05 (1·00-1·09) | 0·05 |
| Excluding meat | 0·99 (0·94-1·04) | 0·63 | 1·05 (1·00-1·09) | 0·04 |
| Excluding miscellaneous animal-based foods | 0·99 (0·94-1·04) | 0·74 |  |  |
| Hazard Ratios with 95% Confidence Intervals (CI) are presented per 10-point increase in the dietary index, reflecting a meaningful increase in adherence. HRs are adjusted for sex, education, ethnicity, BMI, physical activity, smoking status, alcohol intake, energy intake, multimorbidity index, polypharmacy, blood pressure medications, blood thinning medications, cholesterol lowering medications, Townsend deprivation index, and number of completed dietary assessments; stratified by age (5-year categories) and region. All-cause mortality models also adjusted for CVD and cancer at baseline; T2DM models also adjusted for CVD and cancer at baseline; CVD models also adjusted for T2DM and cancer at baseline; cancer models also adjusted for CVD and T2DM at baseline, menopausal status and menopause hormone treatment. For high-UPF uPDI analyses, models also adjusted intake of fruit, nuts, animal-fat, fruit juice and eggs. For low-UPF uPDI, models also adjusted for intake of sugar-sweetened beverages, sweets and desserts and miscellaneous animal-based foods.  P-trend is for linear trend.  *Abbreviations: UPF, ultra-processed food; uPDI, unhealthful plant-based diet index; BMI, Body Mass Index; HR, hazard ratio; CI, confidence interval; T2DM, type 2 diabetes mellitus; CVD, cardiovascular disease.* | | | | |

| **Table S15.** **Sensitivity analysis showing hazard ratios (95% confidence intervals) of all-cause mortality (n=124,791), T2DM (n=119,630), CVD (n=118,874), and cancer (n=112,205) across sex-specific quartiles (Q) of high-ultra-processed and low-ultra-processed hPDI and uPDI, further adjusting for a Modified Nutrient Quality Index** | | | | | | | | | | |
| --- | --- | --- | --- | --- | --- | --- | --- | --- | --- | --- |
|  | **High-UPF hPDI** | | | | P-trend | **Low-UPF hPDI** | | | | P-trend |
|  | Q1 | Q2 | Q3 | Q4 |  | Q1 | Q2 | Q3 | Q4 |  |
| **All-cause mortality** |  |  |  |  |  |  |  |  |  |  |
| Cases/total | 1,799/37,692 | 1,354/30,041 | 1,504/32,670 | 1,123/24,388 |  | 1,474/32,136 | 1,656/35,856 | 1,422/28,767 | 1,228/28,032 |  |
| HR (95% CI) | 1·00^a^ | 0·92 (0·86-0·99) | 0·91 (0·85-0·98) | 0·95 (0·87-1·03) | 0·03 | 1·00^a^ | 0·98 (0·92-1·06) | 0·99 (0·92-1·06) | 0·93 (0·86-1·01) | 0·03 |
| **T2DM** |  |  |  |  |  |  |  |  |  |  |
| Cases/total | 1,253/35,926 | 795/28,894 | 823/31,358 | 549/23,452 |  | 1,042/30,862 | 938/34,466 | 814/27,506 | 626/26,796 |  |
| HR (95% CI) | 1·00^a^ | 0·91 (0·83-1·00) | 0·90 (0·82-1·00) | 0·88 (0·79-0·99) | 0·001 | 1·00^a^ | 0·80 (0·73-0·88) | 0·83 (0·75-0·91) | 0·71 (0·64-0·79) | <0·001 |
| **CVD** |  |  |  |  |  |  |  |  |  |  |
| Cases/total | 1,839/35,892 | 1,464/28,639 | 1,633/31,070 | 1,142/23,273 |  | 1,648/32,999 | 1,510/30,894 | 1,533/27,677 | 1,387/27,304 |  |
| HR (95% CI) | 1·00^a^ | 0·97 (0·90-1·04) | 0·93 (0·87-1·00) | 0·89 (0·82-0·97) | 0·002 | 1·00^a^ | 1·02 (0·95-1·09) | 1·01 (0·94-1·08) | 0·99 (0·91-1·06) | 0·50 |
| **Cancer** |  |  |  |  |  |  |  |  |  |  |
| Cases/total | 2,793/34,046 | 2,225/26,939 | 2,585/29,350 | 1,834/21,870 |  | 2,547/31,093 | 2,439/28,932 | 2,355/26,300 | 2,096/25,880 |  |
| HR (95% CI) | 1·00^a^ | 0·96 (0·91-1·02) | 0·98 (0·93-1·04) | 0·95 (0·89-1·02) | 0·26 | 1·00^a^ | 1·05 (0·99-1·11) | 1·05 (0·99-1·11) | 0·98 (0·92-1·04) | 0·32 |
|  | **High-UPF uPDI** | | | | P-trend | **Low-UPF uPDI** | | | | P-trend |
|  | Q1 | Q2 | Q3 | Q4 |  | Q1 | Q2 | Q3 | Q4 |  |
| **All-cause mortality** |  |  |  |  |  |  |  |  |  |  |
| Cases/total | 1,650/36,207 | 1,508/33,596 | 1,141/24,487 | 1,481/30,501 |  | 1,703/35,354 | 1,419/31,413 | 1,380/29,987 | 1,278/28,037 |  |
| HR (95% CI) | 1·00^a^ | 1·00 (0·93-1·07) | 0·98 (0·91-1·06) | 1·07 (1·00-1·16) | 0·05 | 1·00^a^ | 1·02 (0·95-1·10) | 1·00 (0·93-1·08) | 1·11 (1·03-1·20) | 0·03 |
| **T2DM** |  |  |  |  |  |  |  |  |  |  |
| Cases/total | 880/34,831 | 834/32,332 | 699/23,449 | 1,007/29,018 |  | 880/33,593 | 976/34,013 | 729/25,024 | 835/27,000 |  |
| HR (95% CI) | 1·00^a^ | 1·01 (0·91-1·11) | 1·04 (0·94-1·15) | 1·12 (1·02-1·23) | 0·01 | 1·00^a^ | 1·14 (1·04-1·26) | 1·19 (1·07-1·32) | 1·31 (1·18-1·45) | <0·001 |
| **CVD** |  |  |  |  |  |  |  |  |  |  |
| Cases/total | 1,733/34,592 | 1,572/32,109 | 1,265/23,198 | 1,508/28,975 |  | 1,912/34,488 | 1,457/30,373 | 1,508/28,459 | 1,201/25,554 |  |
| HR (95% CI) | 1·00^a^ | 1·00 (0·93-1·07) | 1·05 (0·97-1·13) | 1·07 (0·99-1·15) | 0·03 | 1·00^a^ | 0·95 (0·88-1·02) | 0·96 (0·90-1·03) | 0·96 (0·89-1·04) | 0·50 |
| **Cancer** |  |  |  |  |  |  |  |  |  |  |
| Cases/total | 2,779/32,438 | 2,643/30,095 | 1,826/22,068 | 2,189/27,604 |  | 2,814/32,400 | 2,390/28,458 | 2,286/27,036 | 1,947/24,311 |  |
| HR (95% CI) | 1·00^a^ | 1·04 (0·99-1·10) | 0·96 (0·91-1·02) | 0·99 (0·93-1·05) | 0·52 | 1·00^a^ | 1·04 (0·98-1·10) | 1·03 (0·97-1·09) | 1·08 (1·01-1·15) | 0·05 |
| Hazard Ratios with 95% Confidence Intervals (CI), adjusted for sex, education, ethnicity, BMI, physical activity, smoking status, alcohol intake, energy intake, multimorbidity index, polypharmacy, blood pressure medications, blood thinning medications, cholesterol lowering medications, Townsend deprivation index, number of completed dietary assessments, and Modified Nutrient Quality Index; stratified by age (5-year categories) and region. All-cause mortality models also adjusted for CVD and cancer at baseline; T2DM models also adjusted for CVD and cancer at baseline; CVD models also adjusted for T2DM and cancer at baseline; cancer models also adjusted for CVD and T2DM at baseline, menopausal status and menopause hormone treatment. For high-UPF hPDI and high-UPF uPDI analyses, models also adjusted intake of fruit, nuts, animal-fat, fruit juice and eggs. For low-UPF hPDI and low-UPF uPDI, models also adjusted for intake of sugar-sweetened beverages, sweets and desserts and miscellaneous animal-based foods.  P-trend is for linear trend.  ^a^Reference categories.  *Abbreviations: Q, quartile; UPF, ultra-processed food; PDI, plant-based diet index; hPDI, healthful plant-based diet index; uPDI, unhealthful plant-based diet index; BMI, Body Mass Index; HR, hazard ratio; CI, confidence interval; T2DM, type 2 diabetes mellitus; CVD, cardiovascular disease.* | | | | | | | | | | |

| **Table S16.** **Hazard ratios (95% confidence intervals) of all-cause mortality (n=124,791), T2DM (n=119,630), CVD (n=118,874), and cancer (n=112,205) across quartiles (Q) of a ratio of low-ultra-processed: high-ultra-processed hPDI and uPDI** | | | | | |
| --- | --- | --- | --- | --- | --- |
|  | **Ratio of low-UPF: high-UPF hPDI** | | | | P-trend |
|  | Q1 | Q2 | Q3 | Q4 |  |
| **All-cause mortality** |  |  |  |  |  |
| Cases/total | 1,438/31,221 | 1,426/31,320 | 1,453/31,553 | 1,463/30,697 |  |
| HR (95% CI) | 1·00^a^ | 1·00 (0·93-1·07) | 1·02 (0·95-1·10) | 1·02 (0·94-1·10) | 0·43 |
| **T2DM** |  |  |  |  |  |
| Cases/total | 854/30,061 | 804/30,116 | 809/29,722 | 953/29,731 |  |
| HR (95% CI) | 1·00^a^ | 0·93 (0·85-1·03) | 0·90 (0·82-1·00) | 0·92 (0·84-1·01) | 0·52 |
| **CVD** |  |  |  |  |  |
| Cases/total | 1,487/30,146 | 1,496/29,403 | 1,494/29,753 | 1,601/29,572 |  |
| HR (95% CI) | 1·00^a^ | 1·03 (0·96-1·11) | 1·04 (0·96-1·11) | 1·06 (0·99-1·14) | 0·05 |
| **Cancer** |  |  |  |  |  |
| Cases/total | 2,399/28,244 | 2,382/28,400 | 2,366/27,842 | 2,290/27,719 |  |
| HR (95% CI) | 1·00^a^ | 1·00 (0·95-1·06) | 1·04 (0·98-1·10) | 1·01 (0·95-1·07) | 0·48 |
|  | **Ratio of low-UPF: high-UPF uPDI** | | | | P-trend |
|  | Q1 | Q2 | Q3 | Q4 |  |
| **All-cause mortality** |  |  |  |  |  |
| Cases/total | 1,572/31,300 | 1,422/32,008 | 1,384/30,801 | 1,402/30,682 |  |
| HR (95% CI) | 1·00^a^ | 0·95 (0·88-1·02) | 0·96 (0·89-1·03) | 0·99 (0·92-1·07) | 0·64 |
| **T2DM** |  |  |  |  |  |
| Cases/total | 905/30,056 | 869/30,353 | 819/29,642 | 827/29,579 |  |
| HR (95% CI) | 1·00^a^ | 1·05 (0·96-1·15) | 1·06 (0·96-1·16) | 1·08 (0·98-1·19) | 0·08 |
| **CVD** |  |  |  |  |  |
| Cases/total | 1,608/29,758 | 1,539/29,819 | 1,513/30,139 | 1,418/29,158 |  |
| HR (95% CI) | 1·00^a^ | 1·01 (0·94-1·08) | 0·99 (0·92-1·06) | 0·94 (0·88-1·02) | 0·11 |
| **Cancer** |  |  |  |  |  |
| Cases/total | 2,376/28,096 | 2,296/28,058 | 2,373/28,357 | 2,392/27,694 |  |
| HR (95% CI) | 1·00^a^ | 1·00 (0·95-1·06) | 1·03 (0·97-1·09) | 1·07 (1·01-1·13) | 0·05 |
| Hazard Ratios with 95% Confidence Intervals (CI), adjusted for sex, education, ethnicity, BMI, physical activity, smoking status, alcohol intake, energy intake, multimorbidity index, polypharmacy, blood pressure medications, blood thinning medications, cholesterol lowering medications, Townsend deprivation index, and number of completed dietary assessments; stratified by age (5-year categories) and region. All-cause mortality models also adjusted for CVD and cancer at baseline; T2DM models also adjusted for CVD and cancer at baseline; CVD models also adjusted for T2DM and cancer at baseline; cancer models also adjusted for CVD and T2DM at baseline, menopausal status and menopause hormone treatment.  P-trend is for linear trend.  ^a^Reference categories.  *Abbreviations: Q, quartile; UPF, ultra-processed food; PDI, plant-based diet index; hPDI, healthful plant-based diet index; uPDI, unhealthful plant-based diet index; BMI, Body Mass Index; HR, hazard ratio; CI, confidence interval; T2DM, type 2 diabetes mellitus; CVD, cardiovascular disease.* | | | | | |

| **Table S17.**  **Hazard ratios (95% confidence intervals) of all-cause mortality (n=124,791), T2DM (n=119,630), CVD (n=118,874), and cancer (n=112,205) across hPDI (10-point increments), stratified by UPF intake** | | | | |
| --- | --- | --- | --- | --- |
|  | **Cases/total** | **hPDI (10-point increments)** | **P-trend** | **P-interaction** |
| **All-Cause Mortality** |  |  |  |  |
| HR (95% CI) |  |  |  | 0·25 |
| UPF Low (<Median) | 2,520/61,978 | 0·95 (0·88-1·02) | 0·13 |  |
| UPF High (≥Median) | 3,260/62,813 | 0·87 (0·82-0·92) | <0·001 |  |
| **T2DM** |  |  |  |  |
| HR (95% CI) |  |  |  | 0·40 |
| UPF Low (<Median) | 1,308/59,775 | 0·87 (0·78-0·95) | 0·004 |  |
| UPF High (≥Median) | 2,112/59,855 | 0·80 (0·74-0·86) | <0·001 |  |
| **CVD** |  |  |  |  |
| HR (95% CI) |  |  |  | 0·61 |
| UPF Low (<Median) | 2,656/59,266 | 0·95 (0·89-1·02) | 0·13 |  |
| UPF High (≥Median) | 3,422/59,608 | 0·93 (0·88-0·98) | 0·01 |  |
| **Cancer** |  |  |  |  |
| HR (95% CI) |  |  |  | 0·30 |
| UPF Low (<Median) | 4,463/55,509 | 0·99 (0·94-1·05) | 0·74 |  |
| UPF High (≥Median) | 4,974/56,696 | 0·96 (0·91-1·00) | 0·06 |  |
| Hazard Ratios with 95% Confidence Intervals (CI) are presented per 10-point increase in the dietary index, reflecting a meaningful increase in adherence. HRs are adjusted for sex, education, ethnicity, BMI, physical activity, smoking status, alcohol intake, energy intake, multimorbidity index, polypharmacy, blood pressure medications, blood thinning medications, cholesterol lowering medications, Townsend deprivation index, and number of completed dietary assessments; stratified by age (5-year categories) and region. All-cause mortality models also adjusted for CVD and cancer at baseline; T2DM models also adjusted for CVD and cancer at baseline; CVD models also adjusted for T2DM and cancer at baseline; cancer models also adjusted for CVD and T2DM at baseline, menopausal status and menopause hormone treatment.  P-interaction values were obtained using likelihood ratio tests, comparing models with and without an interaction term between the PDI exposure (quartiles) and UPF intake (binary).  P-trend is for linear trend.  *Abbreviations: UPF, ultra-processed food; PDI, plant-based diet index; hPDI, healthful plant-based diet index; BMI, Body Mass Index; HR, hazard ratio; CI, confidence interval; T2DM, type 2 diabetes mellitus; CVD, cardiovascular disease.* | | | | |

| **Table S18.**  **Hazard ratios (95% confidence intervals) of all-cause mortality (n=124,791), T2DM (n=119,630), CVD (n=118,874), and cancer (n=112,205) across uPDI (10-point increments), stratified by UPF intake** | | | | |
| --- | --- | --- | --- | --- |
|  | **Cases/total** | **uPDI (10-point increments)** | **P-trend** | **P-interaction** |
| **All-Cause Mortality** |  |  |  |  |
| HR (95% CI) |  |  |  | 0·55 |
| UPF Low (<Median) | 2,520/61,978 | 1·08 (1·00-1·16) | 0·05 |  |
| UPF High (≥Median) | 3,260/62,813 | 1·12 (1·06-1·19) | <0·001 |  |
| **T2DM** |  |  |  |  |
| HR (95% CI) |  |  |  | 0·88 |
| UPF Low (<Median) | 1,308/59,775 | 1·15 (1·04-1·27) | 0·006 |  |
| UPF High (≥Median) | 2,112/59,855 | 1·22 (1·13-1·31) | <0·001 |  |
| **CVD** |  |  |  |  |
| HR (95% CI) |  |  |  | 0·58 |
| UPF Low (<Median) | 2,656/59,266 | 1·03 (0·96-1·10) | 0·40 |  |
| UPF High (≥Median) | 3,422/59,608 | 1·04 (0·99-1·11) | 0·15 |  |
| **Cancer** |  |  |  |  |
| HR (95% CI) |  |  |  | 0·81 |
| UPF Low (<Median) | 4,463/55,509 | 1·04 (0·98-1·09) | 0·21 |  |
| UPF High (≥Median) | 4,974/56,696 | 1·02 (0·98-1·07) | 0·35 |  |
| Hazard Ratios with 95% Confidence Intervals (CI) are presented per 10-point increase in the dietary index, reflecting a meaningful increase in adherence. HRs are adjusted for sex, education, ethnicity, BMI, physical activity, smoking status, alcohol intake, energy intake, multimorbidity index, polypharmacy, blood pressure medications, blood thinning medications, cholesterol lowering medications, Townsend deprivation index, and number of completed dietary assessments; stratified by age (5-year categories) and region. All-cause mortality models also adjusted for CVD and cancer at baseline; T2DM models also adjusted for CVD and cancer at baseline; CVD models also adjusted for T2DM and cancer at baseline; cancer models also adjusted for CVD and T2DM at baseline, menopausal status and menopause hormone treatment.  P-interaction values were obtained using likelihood ratio tests, comparing models with and without an interaction term between the PDI exposure (quartiles) and UPF intake (binary).  P-trend is for linear trend.  *Abbreviations: UPF, ultra-processed food; PDI, plant-based diet index; uPDI, unhealthful plant-based diet index; BMI, Body Mass Index; HR, hazard ratio; CI, confidence interval; T2DM, type 2 diabetes mellitus; CVD, cardiovascular disease.* | | | | |

| **Table S19. Sensitivity analysis showing hazard ratios (95% confidence intervals) of all-cause mortality (n=124,791), T2DM (n=119,630), CVD (n=118,874), and cancer (n=112,205) across high-ultra-processed and low-ultra-processed hPDI and uPDI (10-point increments), stratified by Sex** | | | | | | | | |
| --- | --- | --- | --- | --- | --- | --- | --- | --- |
|  | **High-UPF hPDI**  **(10-point increments)** | | | **Low-UPF hPDI**  **(10-point increments)** | | |  |  |
|  | **Cases/total** | **HR (95% CI)** | **P-trend** | **P-interaction** | **HR (95% CI)** | **P-trend** | **P-interaction** |  |
| **All-cause mortality** |  |  |  |  |  |  |  |  |
| Male | 3,350/55,127 | 0·90 (0·83-0·98) | 0·01 | 0·94 | 0·96 (0·89-1·03) | 0·20 | 0·88 |  |
| Female | 2,430/69,664 | 0·91 (0·83-1·01) | 0·08 |  | 0·91 (0·84-0·99) | 0·03 |  |  |
| **T2DM** |  |  |  |  |  |  |  |  |
| Male | 2,004/51,973 | 0·85 (0·76-0·94) | 0·001 | 0·88 | 0·86 (0·79-0·93) | <0·001 | 0·69 |  |
| Female | 1,416/67,657 | 0·91 (0·80-1·03) | 0·15 |  | 0·76 (0·68-0·84) | <0·001 |  |  |
| **CVD** |  |  |  |  |  |  |  |  |
| Male | 3,727/51,065 | 0·89 (0·82-0·95) | 0·001 | 1·00 | 0·96 (0·90-1·02) | 0·17 | 0·15 |  |
| Female | 2,351/67,809 | 0·95 (0·85-1·05) | 0·29 |  | 1·02 (0·95-1·11) | 0·57 |  |  |
| **Cancer** |  |  |  |  |  |  |  |  |
| Male | 5,047/50,498 | 0·94 (0·88-1·00) | 0·04 | 0·67 | 0·98 (0·93-1·03) | 0·41 | 0·58 |  |
| Female | 4,390/61,707 | 1·00 (0·93-1·08) | 1·00 |  | 0·96 (0·91-1·02) | 0·20 |  |  |
|  | **High-UPF uPDI**  **(10-point increments)** | | | **Low-UPF uPDI**  **(10-point increments)** | | |  |  |
|  | **Cases/total** | **HR (95% CI)** | **P-trend** | **P-interaction** | **HR (95% CI)** | **P-trend** | **P-interaction** |  |
| **All-cause mortality** |  |  |  |  |  |  |  |  |
| Male | 3,350/55,127 | 1·09 (1·00-1·17) | 0·04 | 0·41 | 1·06 (0·99-1·14) | 0·09 | 0·56 |  |
| Female | 2,430/69,664 | 1·07 (0·97-1·18) | 0·18 |  | 1·11 (1·02-1·20) | 0·02 |  |  |
| **T2DM** |  |  |  |  |  |  |  |  |
| Male | 2,004/51,973 | 1·08 (0·98-1·19) | 0·15 | 0·15 | 1·15 (1·05-1·26) | 0·003 | 0·59 |  |
| Female | 1,416/67,657 | 1·16 (1·02-1·31) | 0·03 |  | 1·24 (1·11-1·38) | <0·001 |  |  |
| **CVD** |  |  |  |  |  |  |  |  |
| Male | 3,727/51,065 | 1·07 (0·99-1·15) | 0·09 | 0·18 | 0·97 (0·90-1·04) | 0·33 | 0·85 |  |
| Female | 2,351/67,809 | 1·09 (0·98-1·20) | 0·11 |  | 1·00 (0·92-1·09) | 0·93 |  |  |
| **Cancer** |  |  |  |  |  |  |  |  |
| Male | 5,047/50,498 | 0·99 (0·92-1·05) | 0·67 | 0·47 | 1·06 (1·00-1·12) | 0·06 | 0·65 |  |
| Female | 4,390/61,707 | 0·98 (0·91-1·06) | 0·66 |  | 1·04 (0·98-1·11) | 0·19 |  |  |
| Hazard Ratios with 95% Confidence Intervals (CI) are presented per 10-point increase in the dietary index, reflecting a meaningful increase in adherence. HRs are adjusted for education, Townsend deprivation index, ethnicity, BMI, physical activity, smoking status, alcohol intake, energy intake, multimorbidity index, polypharmacy, blood pressure medications, blood thinning medications, cholesterol lowering medications, and number of completed dietary assessments. All-cause mortality models also adjusted for CVD and cancer at baseline; T2DM models also adjusted for CVD and cancer at baseline; CVD models also adjusted for T2DM and cancer at baseline; cancer models also adjusted for CVD and T2DM at baseline, menopausal status and menopause hormone treatment. For high-UPF hPDI and high-UPF uPDI analyses, models also adjusted intake of fruit, nuts, animal-fat, fruit juice and eggs. For low-UPF hPDI and low-UPF uPDI, models also adjusted for intake of sugar-sweetened beverages, sweets and desserts and miscellaneous animal-based foods  P-interaction values were obtained using likelihood ratio tests, comparing models with and without an interaction term between the PDI exposure (quartiles) and Sex.  P-trend is for linear trend.  *Abbreviations: Q, quartile; UPF, ultra-processed food; PDI, plant-based diet index; hPDI, healthful plant-based diet index; uPDI, unhealthful plant-based diet index; BMI, Body Mass Index; HR, hazard ratio; CI, confidence interval; T2DM, type 2 diabetes mellitus; CVD, cardiovascular disease.* | | | | | | | | |

| **Table S20. Hazard ratios (95% confidence intervals) of mortality across sex-specific quartiles (Q) of high-ultra-processed and low-ultra-processed hPDI and uPDI, excluding deaths occurring within 1 year of the last dietary assessment** | | | | | | | | | | |
| --- | --- | --- | --- | --- | --- | --- | --- | --- | --- | --- |
|  | **High-UPF hPDI** | | | | P-trend | **Low-UPF hPDI** | | | | P-trend |
|  | Q1 | Q2 | Q3 | Q4 |  | Q1 | Q2 | Q3 | Q4 |  |
| **All-cause mortality** |  |  |  |  |  |  |  |  |  |  |
| Cases/total | 1,678/37,571 | 1,252/29,939 | 1,397/32,563 | 1,060/24,325 |  | 1,359/32,021 | 1,539/35,739 | 1,330/28,675 | 1,159/27,963 |  |
| HR (95% CI) | 1·00^a^ | 0·91 (0·84-0·98) | 0·90 (0·83-0·97) | 0·94 (0·86-1·02) | 0·01 | 1·00^a^ | 0·99 (0·92-1·06) | 0·99 (0·92-1·07) | 0·93 (0·86-1·01) | 0·03 |
|  | **High-UPF uPDI** | | | | P-trend | **Low-UPF uPDI** | | | | P-trend |
|  | Q1 | Q2 | Q3 | Q4 |  | Q1 | Q2 | Q3 | Q4 |  |
| **All-cause mortality** |  |  |  |  |  |  |  |  |  |  |
| Cases/total | 1,551/36,108 | 1,394/33,482 | 1,062/24,408 | 1,380/30,400 |  | 1,617/35,268 | 1,325/31,319 | 1,277/29,884 | 1,168/27,927 |  |
| HR (95% CI) | 1·00^a^ | 0·99 (0·92-1·06) | 0·99 (0·91-1·07) | 1·09 (1·01-1·17) | 0·02 | 1·00^a^ | 1·02 (0·94-1·09) | 1·00 (0·92-1·08) | 1·11 (1·02-1·20) | 0·04 |
| Hazard Ratios with 95% Confidence Intervals (CI), adjusted for sex, education, ethnicity, BMI, physical activity, smoking status, alcohol intake, energy intake, multimorbidity index, polypharmacy, blood pressure medications, blood thinning medications, cholesterol lowering medications, Townsend deprivation index, number of completed dietary assessments, CVD and cancer at baseline; stratified by age (5-year categories) and region. For high-UPF hPDI and high-UPF uPDI analyses, models also adjusted intake of fruit, nuts, animal-fat, fruit juice and eggs. For low-UPF hPDI and low-UPF uPDI, models also adjusted for intake of sugar-sweetened beverages, sweets and desserts and miscellaneous animal-based foods.  P-trend is for linear trend.  ^a^Reference categories.  *Abbreviations: Q, quartile; UPF, ultra-processed food; PDI, plant-based diet index; hPDI, healthful plant-based diet index; uPDI, unhealthful plant-based diet index; BMI, Body Mass Index; HR, hazard ratio; CI, confidence interval; CVD, cardiovascular disease.* | | | | | | | | | | |

| **Table S21. Hazard ratios (95% confidence intervals) of mortality across sex-specific quartiles (Q) of high-ultra-processed and low-ultra-processed hPDI and uPDI, with follow-up restricted to before 2020 (pre-COVID-19 pandemic)** | | | | | | | | | | |
| --- | --- | --- | --- | --- | --- | --- | --- | --- | --- | --- |
|  | **High-UPF hPDI** | | | | P-trend | **Low-UPF hPDI** | | | | P-trend |
|  | Q1 | Q2 | Q3 | Q4 |  | Q1 | Q2 | Q3 | Q4 |  |
| **All-cause mortality** |  |  |  |  |  |  |  |  |  |  |
| Cases/total | 1,285/37,692 | 965/30,041 | 1,086/32,670 | 797/24,388 |  | 1,048/32,136 | 1,207/35,856 | 991/28,767 | 887/28,032 |  |
| HR (95% CI) | 1·00^a^ | 0·91 (0·84-0·99) | 0·90 (0·83-0·98) | 0·90 (0·82-1·00) | 0·008 | 1·00^a^ | 0·99 (0·91-1·08) | 0·94 (0·86-1·03) | 0·92 (0·84-1·00) | 0·02 |
|  | **High-UPF uPDI** | | | | P-trend | **Low-UPF uPDI** | | | | P-trend |
|  | Q1 | Q2 | Q3 | Q4 |  | Q1 | Q2 | Q3 | Q4 |  |
| **All-cause mortality** |  |  |  |  |  |  |  |  |  |  |
| Cases/total | 1,155/36,207 | 1,058/33,596 | 843/24,487 | 1,077/30,501 |  | 1,179/35,354 | 1,018/31,413 | 997/29,987 | 939/28,037 |  |
| HR (95% CI) | 1·00^a^ | 1·01 (0·93-1·10) | 1·05 (0·96-1·15) | 1·14 (1·04-1·24) | 0·002 | 1·00^a^ | 1·06 (0·97-1·15) | 1·04 (0·95-1·14) | 1·18 (1·08-1·30) | 0·004 |
| Hazard Ratios with 95% Confidence Intervals (CI), adjusted for sex, education, ethnicity, BMI, physical activity, smoking status, alcohol intake, energy intake, multimorbidity index, polypharmacy, blood pressure medications, blood thinning medications, cholesterol lowering medications, Townsend deprivation index, number of completed dietary assessments, CVD and cancer at baseline; stratified by age (5-year categories) and region. For high-UPF hPDI and high-UPF uPDI analyses, models also adjusted intake of fruit, nuts, animal-fat, fruit juice and eggs. For low-UPF hPDI and low-UPF uPDI, models also adjusted for intake of sugar-sweetened beverages, sweets and desserts and miscellaneous animal-based foods.  P-trend is for linear trend.  ^a^Reference categories.  *Abbreviations: Q, quartile; UPF, ultra-processed food; PDI, plant-based diet index; hPDI, healthful plant-based diet index; uPDI, unhealthful plant-based diet index; BMI, Body Mass Index; HR, hazard ratio; CI, confidence interval; CVD, cardiovascular disease.* | | | | | | | | | | |
